# A Review on Rhenium Disulfide: Synthesis Approaches, Optical Properties, and Applications in Pulsed Lasers

**DOI:** 10.3390/nano11092367

**Published:** 2021-09-12

**Authors:** Mahmoud Muhanad Fadhel, Norazida Ali, Haroon Rashid, Nurfarhana Mohamad Sapiee, Abdulwahhab Essa Hamzah, Mohd Saiful Dzulkefly Zan, Norazreen Abd Aziz, Norhana Arsad

**Affiliations:** Department of Electrical, Electronic and Systems Engineering, Faculty of Engineering and Built Environment, Universiti Kebangsaan Malaysia, UKM, Bangi 43600, Malaysia; P97209@siswa.ukm.edu.my (M.M.F.); P96194@siswa.ukm.edu.my (N.A.); haroon@ukm.edu.my (H.R.); P103517@siswa.ukm.edu.my (N.M.S.); P97926@siswa.ukm.edu.my (A.E.H.); saifuldzul@ukm.edu.my (M.S.D.Z.); norazreen@ukm.edu.my (N.A.A.)

**Keywords:** saturable absorbers, Rhenium disulfide, pulsed lasers, mode-locking, Q-switching, 2D TMD

## Abstract

Rhenium Disulfide (ReS_2_) has evolved as a novel 2D transition-metal dichalcogenide (TMD) material which has promising applications in optoelectronics and photonics because of its distinctive anisotropic optical properties. Saturable absorption property of ReS_2_ has been utilized to fabricate saturable absorber (SA) devices to generate short pulses in lasers systems. The results were outstanding, including high-repetition-rate pulses, large modulation depth, multi-wavelength pulses, broadband operation and low saturation intensity. In this review, we emphasize on formulating SAs based on ReS_2_ to produce pulsed lasers in the visible, near-infrared and mid-infrared wavelength regions with pulse durations down to femtosecond using mode-locking or Q-switching technique. We outline ReS_2_ synthesis techniques and integration platforms concerning solid-state and fiber-type lasers. We discuss the laser performance based on SAs attributes. Lastly, we draw conclusions and discuss challenges and future directions that will help to advance the domain of ultrafast photonic technology.

## 1. Introduction

Ultrafast laser technology pertains to the creation, amplification and operation of ultrashort pulses with periods ranging from nanosecond to picosecond and femtosecond. Numerous distinct characteristics of pulsed lasers such as greater peak intensity, broad spectrum and fast temporal resolution [1] have driven a wide-range of uses such as laser welding and drilling [2], ultrafast spectroscopy [3,4] and frequency comb metrology [5,6]. Furthermore, pulsed lasers based on intensity modulation have proven their significant efficiency within a wide variety of distributed optical fiber sensors (DOFS) [7,8,9,10,11]. These kinds of lasers could be produced either by Q-switching or mode-locking methods, which are categorized into passive or active modulation methodologies. Typically, the Q-switching method can provide immense short-duration pulses in the microsecond to nanosecond range by regulating cavity loss frequently using a kilohertz-range frequency. On the other hand, the mode-locking method [12] comprising a concurrent in-phase lock of all the longitudinal modes in the cavity is frequently employed for producing extremely-short pulses ranging from a few femtoseconds to several picoseconds. Furthermore, the mode-locked pulse repetition rate is quite higher compared to the Q-switching repetition rate [13].

The active modulation technique relies on an optical modulator with an externally-applied modulating signal; the modulator is usually based on the electro-optic or acousto-optic effect [1,14]. Even though this methodology is quite robust, it fails to create very short pulses because of the inadequate bandwidth of active modulators. Conversely, passive modulation designs are in a position to offer quite shorter pulses, as the SA (saturable absorber) with a brief recovery time can regulate the cavity loss much quicker compared to an electronic optical modulator [15].

The saturable absorbers in passive Q-switching and mode-locking approaches could be generally split into two classes: real SAs, materials which demonstrate an intrinsic nonlinear drop in absorption with growing light intensity; and artificial SAs, devices which manipulate nonlinear effects, such as nonlinear polarization rotation (NPR) and nonlinear amplifying loop mirror (NALM), to simulate the action of a real SA by provoking an intensity-dependent transmission [16]. Nevertheless, NPR and NALM face several issues, such as high saturable threshold power and polarization sensitivity [17].

**Figure 1:**
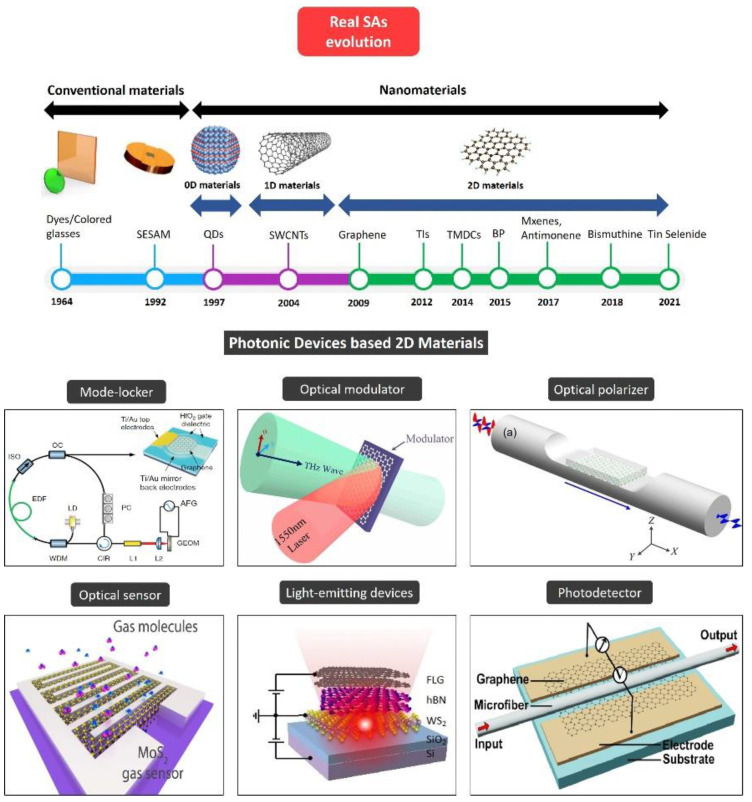
The evolution of real saturable absorber technologies and their applications as photonic devices. Reprinted with permission from ref. [24]. Copyright 2018 John Wiley and Sons. Reprinted under the terms of a Creative Commons Attribution 4.0 International License from ref. [25]. Copyright 2014 Springer Nature. Reprinted under the terms of a Creative Commons Attribution 4.0 International License from ref. [26]. Copyright 2016 Springer Nature. Reprinted under the terms of a Creative Commons Attribution 4.0 International License from ref. [27]. Copyright 2015 Springer Nature. Adapted with permission from ref. [28]. Copyright 2017 American Chemical Society. Reprinted under the terms of a Creative Commons Attribution 4.0 International License from ref. [29]. Copyright 2015 The Optical Society.

The advancements in microfabrication and material science have driven a growing number of materials which could be utilized as real SAs. Figure 1 depicts the progression of real SA technologies and their applications as photonic devices. In 1964, “reversibly bleachable” dye [18] and colored glass filters [19] were used for the first-ever presentation of SA-based pulse generation by Q-switching a ruby laser, just four years after Maiman demonstrated laser operation [20]. Reversibly bleachable dyes were extensively deployed to mode-lock lasers, driving the first exhibition of continuous-wave (CW) mode locking [21]. Later in 1983, ion-doped fiber has emerged as gain medium, and unstable mode-locking of a Nd:Fiber laser was reported using a dye SA [22]. It was quite challenging to create steady mode-locked pulses among fiber lasers, till the exhibition of semiconductor saturable absorber mirror (SESAM) in 1992 [23], a discovery which facilitated the first display of a passively mode-locked fiber laser (Nd:YLF) minus Q-switching instabilities. However, producing SESAMs typically comprises sophisticated and highly specialized instruments. Post-growth ion insertion or the uses of low temperature growth are employed for minimizing device response time. Additionally, a typical SESAM-based instrument operates in a narrow wavelength band (<100 nm) [1].

Unfortunately, such traditional SA materials have certain disadvantages, and therefore are not able to fulfill the main SA requirements, such as fast response time, strong nonlinearity, low loss, broad bandwidth, greater power handling and low costs [30]. SA material with nanometer-scale dimensionality may offer pronounced optoelectronic characteristics and powerful quantum confinement [31]. In 1997, zero-dimensional quantum dots (QDs) were used for pulse generation [32]. This presentation led to widespread interest in nanomaterial SAs. One-dimensional single-walled carbon nanotubes (SWCNTs) [33] and two-dimensional graphene [34] were regarded as potent materials for creating ultrafast pulses in the sub-picosecond range [30]. Usually, nanotubes with distinct diameters and chirality are tough to synthesize [35].

**Figure 2:**
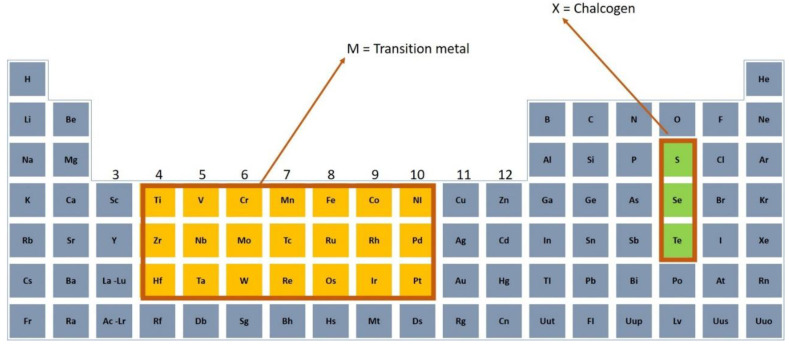
Periodic table showing TMDs materials with its components of transition metals M and three chalcogen X elements.

Apart from graphene, which was originally found in 2004 [36] with zero-bandgap structure, there are a broader category of novel 2D materials such as topological insulators (TIs) [37,38], transition metal dichalcogenides (TMDs) [39,40], black phosphorus (BP) [41,42], MXenes [43], antimonene [44,45], bismuthine [46,47,48] and tin selenide [49]. Nowadays, 2D materials are a preference for research and use in nearly each domain of science and engineering, especially TMDs which demonstrate adaptable chemistry. TMDs having MX_2_ chemical formula typically comprise a plane having hexagonally-placed transition metal atoms M (groups 4–10) in Figure 2 placed between two chalcogen atom-based hexagonal planes X (e.g., S, Se, Te). The M-X bonds within layers are mostly covalent, while weak Van der Waals forces hold the sandwiched layers [50]. TMDs have been acknowledged since the 60 s, and a set of 40 TMDs and their elementary attributes was reviewed in 1969 [51].

Recently, Rhenium disulfide (ReS_2_) in group 7 has been drawing the highest attention recently due to its uncommon electro-optical, structural and chemical attributes [52]. Contrasting to group 6 TMDs which steadied in extremely symmetric 2H structures, ReS_2_ possesses a distinctive distorted 1T structure that renders an in-plane anisotropy to its physical attributes [53]. Furthermore, because of the exceptionally weak interlayer coupling, bulk ReS_2_ functions as vibrational and electronically decoupled monolayers, causing a noticeable layer-independent character in different attributes [54]. Such unique attributes of ReS_2_ have encouraged several evolving applications such as catalysis, energy storage, optoelectronic devices and sensing [55,56,57]. Considering the strong light absorbance in a wide wavelength band, ReS_2_ material is potentially useful for optical modulation as required for pulsed lasers using SAs [58,59].

Report about the nonlinear saturable absorption response of ReS_2_ beyond its bandgap were first appeared in the literature in 2017 [60]. Yudong Cui et al. studied the optical response of ReS_2_ at around 1550 nm with the help of D-shaped fiber. The material was produced via chemical vapor deposition (CVD) method. However, this production method is complex and expensive. Later, much simpler and low-cost methods were used to synthesize ReS_2_ such as mechanical exfoliation (ME) [61] and liquid phase exfoliation (LPE) [62]. Along with saturable absorption response, investigating the large third-order nonlinear effect of ReS_2_ has enabled the generation of harmonic mode-locking lasers with high repetition rates pulses. Most of the reported ReS_2_-based SAs are made of solution using LPE method and deposited on fiber platforms such as the fiber ferrule [63,64,65] and microfiber [66,67] or on substrates such as sapphire [62,68,69,70] and quartz [71].

Here, we review the latest developments on generating short laser pulses using saturable absorbers based on ReS_2_. In Section 2 we mainly introduce the fundamental structure of ReS_2_ and its optical properties. Followed by the material fabrication methods and integration in free-space and fiber laser systems. In the last section we discussed the laser performance based on several parameters such as material thickness, SA modulation depth and optical damage threshold. On the basis of these developments, a conclusion and point of view for new prospective opportunities for ultrafast photonic technologies are emphasized.

## 2. Structure of ReS_2_ and Its Synthesis Methods

### 2.1. Rhenium Disulfide (ReS_2_)

**Figure 3:**
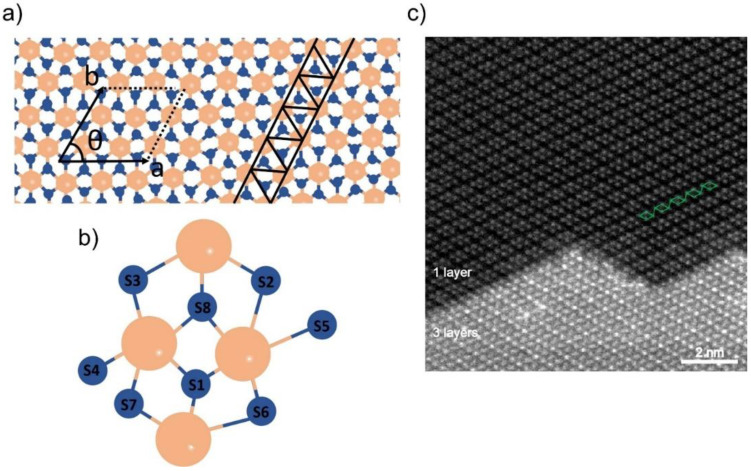
(**a**) Atomic structure of a monolayer ReS_2_. Unit cell and Re chains are indicated. (**b**) Top view of the ReS_2_ monolayer shown by unit cell atoms. (**c**) Low magnification ADF image of ReS_2_. The upper part is single-layer with the diamond-shape (DS) phase structure, while the lower part is three-layer stacking. Reprinted with permission from ref. [73]. Copyright 2015 American Chemical Society.

Noddack, Tacke and Berg [72] were the first to discover rhenium in 1925, the last steady element in the periodic table [53]. The atomic arrangement of a ReS_2_ layer may be understood as a distorted 1T arrangement. The layer comprises a zigzag Re-Re chains along the b-axis of the lattice (Figure 3a) [54]. Metal-metal bonding causes doubling on the ReS_2_ unit cell, which, therefore, comprises eight S and four Re atoms (Figure 3b). Figure 3c depicts a less zoomed ReS_2_ annular dark field (ADF) picture showing tri- and monolayer regions on the flakes [73]. The ADF image clearly shows single layer regions comprising four Re atoms forming diamond-shaped links (green line).

**Figure 4:**
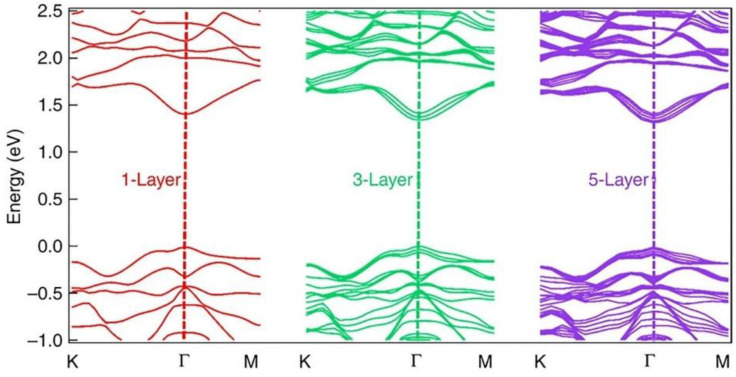
Ab initio calculated electronic band structure of monolayer, trilayer and five-layer ReS_2_ indicating band gaps of 1.44, 1.4 and 1.35 eV, respectively. Reprinted under the terms of a Creative Commons Attribution 4.0 International License from ref. [74]. Copyright 2015 Springer Nature.

ReS_2_ has a noteworthy aspect: the band structures for monolayer, trilayer and five-layer ReS2 are similar (Figure 4), indicating the nature of ReS_2_ of maintaining a direct-bandgap [74]. On the other hand, several TMDs have direct and indirect bandgaps in monolayer and bulk forms, respectively [54]. Bandgap characteristics can cause thickness to affect TMD properties extensively. Furthermore, based on the strain as well as ribbon width, the bandgap and electronic properties of mono-layered TMDs will alter greatly [75,76]. Ab initio calculations indicate that ReS_2_ bandgap does not change significantly when the thickness is increased from monolayer (1.44 eV), trilayer (1.4 eV) to five-layer (1.35 eV) [74]. This distinct response can be attributed to the absence of interlayer registry and weak interlayer connection caused by Peierls distortion of the ReS_2_ 1T arrangement [54]. Additionally, the comparability of optical and electrical characteristics of single-layer and bulk ReS_2_ indicates that the material could offer a base to evaluate 2D system mesoscopic physics without facing the challenge of producing thick monolayer flakes with substantial area [67].

**Figure 5:**
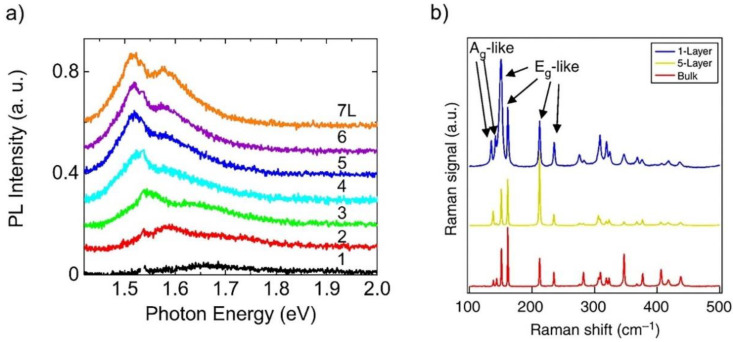
(**a**) Photoluminescence (PL) spectrum of ReS_2_ flakes with different number of layers. Reprinted with permission from ref. [78]. Copyright 2018 Applied Physics Letters. (**b**) 18 Raman modes observed on monolayer, five-layer and bulk ReS_2_. Six labelled Raman modes include two low frequency Ag-like modes corresponding to the out-of-plane vibrations of Re atoms and four Eg-like modes corresponding to the in-plane vibrations of Re atoms. The rest 12 higher frequency Raman modes are vibrations mainly from lighter S atoms. Reprinted under the terms of a Creative Commons Attribution 4.0 International License from ref. [74]. Copyright 2015 Springer Nature.

ReS_2_ in bulk form has anisotropic optical characteristics that are recorded [77]. The present emphasis is on the material comprising few layers and the monolayer structure [69]. Figure 5a depicts the photoluminescence (PL) spectrum of ReS_2_ flakes with diverse quantity of layers [78]. Thinning of ReS_2_ material form 7-layer to monolayer thickness is associated with a reduction in PL intensity. However, the red-shift associated with the peak position is insignificant, indicating little dependence on layer count. This observation is in agreement with theoretical bandgap calculations of ReS_2_ [52].

The ReS_2′_s Raman response is shown in many research works [79,80,81]. Due to the low symmetry of ReS_2_, a higher number of Raman modes could be seen as against the traditional TMDs. Additionally, the frequency shift for every Raman mode indicates minute changes concerning monolayer to bulk ReS_2_, as shown in Figure 5b; the figure indicates 18 Raman modes corresponding to bulk, five-layer and monolayer ReS_2_ [74]. There are six Raman modes with labels; these comprise two Ag-like low-frequency modes (identified at 136.8 and 144.5 cm^−1^) specific to out-plane Re atom vibrations. Furthermore, four Eg-like modes are observed (identified at 153.6, 163.4, 218.2 and 238.1 cm^−1^) specific to in-plane Re atom vibrations. The remaining Raman modes possess high frequency and are specific to relatively light S atoms [80]. Thus, one can determine the thickness of few-layer ReS_2_ by analyzing the Raman peak positions, much akin to the approach utilized for MoS_2_ [82].

### 2.2. Synthesis Techniques

**Figure 6:**
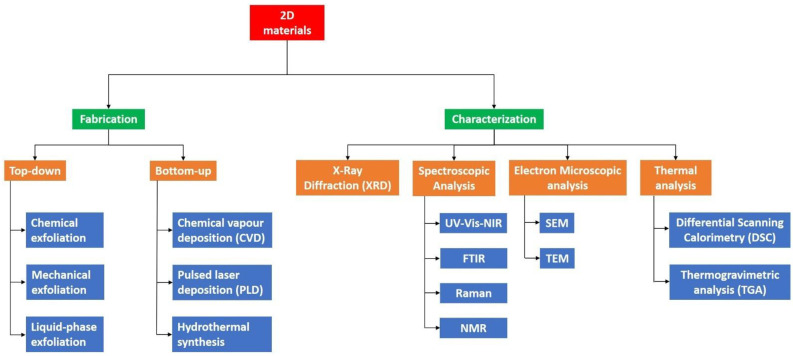
Typical fabrication and characterization methods for 2D TMD materials.

The systematic fabrication of 2D ReS_2_ with suitable size, thickness, morphology and crystal quality is substantially important for the exploration of their electronic, optical and thermal properties for prospective applications. Rhenium (Re) retains one of the highest melting points (~3180 °C) among all transition metals whereas disulfide is comparatively low (~155 °C). The large difference in melting points makes it considerably challenging to grow ReS_2_ thin films. This section summarizes the various fabrication techniques of ReS_2_. First part highlights the top-down synthesis approach which comprises of mechanical and liquid phase exfoliations. Subsequent part discusses bottom-up techniques which includes the physical vapor deposition (PVD), chemical vapor transport (CVT) and chemical vapor deposition (CVD) techniques [52]. Depending on the applications’ need, various characterizations can be performed for the 2D TMD ReS_2_ films. Figure 6 encapsulates the various fabrication and characterization techniques commonly used for 2D TMD ReS_2_.

#### 2.2.1. Mechanical Exfoliation (ME)

**Figure 7:**
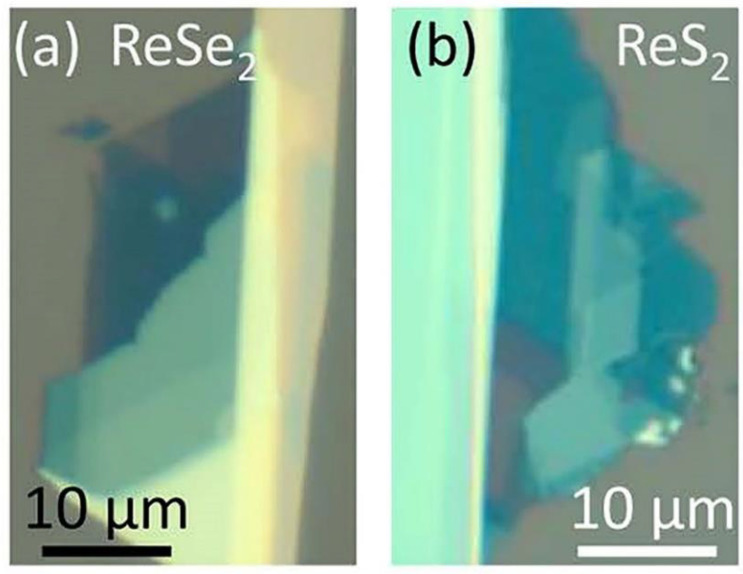
Optical image of a N-layer (**a**) ReSe_2_ and (**b**) ReS_2_ crystal. Reproduced with permission from Ref. [88]. Copyright 2016, American Chemical Society.

Mechanical exfoliation has an outstanding influence on study and use of 2D materials on their fundamental characteristics. 2D graphene films were first acquired by Novoselov et al. [36] using the scotch tape method scientifically known as the mechanical exfoliation technique. It offers crucial access to high-quality flakes with good mechanical and electrical characteristics in spite of the inexpensive and coarse method [83,84,85,86]. A single crystal of bulk material is affixed to the adhesive side of the scotch tape and subsequently an additional piece of tape is positioned on the opposite side of the bulk material. Afterwards, both pieces of tape are peeled for numerous times. A clean and flat substrate, usually SiO_2_/Si (300 nm) is then used to affix the freshly sliced thin flake from the scotch tape. Similarly, mono or few layers can be obtained and transferred to the targeted substrate [52]. A highly responsive phototransistors using few layers of ReS_2_ is demonstrated by Liu et al. [87] using mechanical exfoliation approach. Figure 7 reveals the usual optical images of ReSe_2_ and ReS_2_ on SiO_2_/Si substrate with various layers exfoliated from the bulk materials [88]. However, the shortcomings of this method cannot be overlooked. It may produce edges and ribbons beside crystallographic directions. In addition, edges, number of layers and morphology are intense, owing to coarse procedure [89].

#### 2.2.2. Liquid Phase Exfoliation (LPE)

Liquid phase exfoliation is an effective technique for exfoliating ReS_2_ nanosheets in large capacity without losing crystal quality. Currently, ReS_2_ exfoliation can be classified into two main types; sonication assisted exfoliation and ion intercalation exfoliation [90]. Hersam et al. has reported the ReS_2_ exfoliation via layer-by-layer isopycnic density gradient ultracentrifugation sorting of high density nanosheets in aqueous surfactant solutions [90]. At first, ReS_2_ powder in deionized water was sonicated with the amphiphilic small molecule surfactant sodium cholate. Subsequently, centrifuged at 7500 RPM to remove the unexfoliated flakes and the supernatant was collected. To precipitate large size ReS_2_ nanosheets, it was further centrifuged at 20,000 RPM. Finally, the comparatively uniform ReS_2_ nanosheets were attained with average thickness of around 3 nm and 50–100 nm of lateral size. A mixed solvent strategy was demonstrated using the Hansen solubility theory to prepare few layers of ReS_2_ nanosheets by exfoliating bulk ReS_2_ in an ethanol-water mixture [91]. It was reported that using a mixture of solvent with 72% deionized water and 28% ethanol for sonication is optimum to efficiently exfoliate large scale nanosheets of ReS_2_ with an average lateral size of 2.3 nm and a thickness of 50–80 nm. Colloidal ReS_2_ nanosheets for antitumor therapy and bioimaging applications using a sonication assisted liquid exfoliation method are also reported by Miao et al. [92].

Another technique to prepare ReS_2_ nanosheets is ion intercalation exfoliation. In this method, the cation, e.g., Na^+^, Li^+^, K^+^, with small ionic radius can easily insert into the interspace of layered bulk crystals. It expand the interspace drastically and weaken the Van der Waals forces between contiguous layers [52]. Another technique concerning the reaction of ReS_2_ powder with lithium borohydride (LiBH_4_) is developed to replace the conventional protocol requiring butyl lithium solution to effectively exfoliate ReS_2_ nanosheets [93]. Nevertheless, the obtained ReS_2_ nanosheets with respect to thickness, and lateral size are polydisperse. A homogeneous monolayer ReS_2_ film at large scale is challenging. Furthermore, certain solvents and reagents may cause contamination. In this perspective, the nanosheets obtained by this technique are more suited to biological and energy conversion applications [52].

#### 2.2.3. Physical Vapor Deposition (PVD)

**Figure 8:**
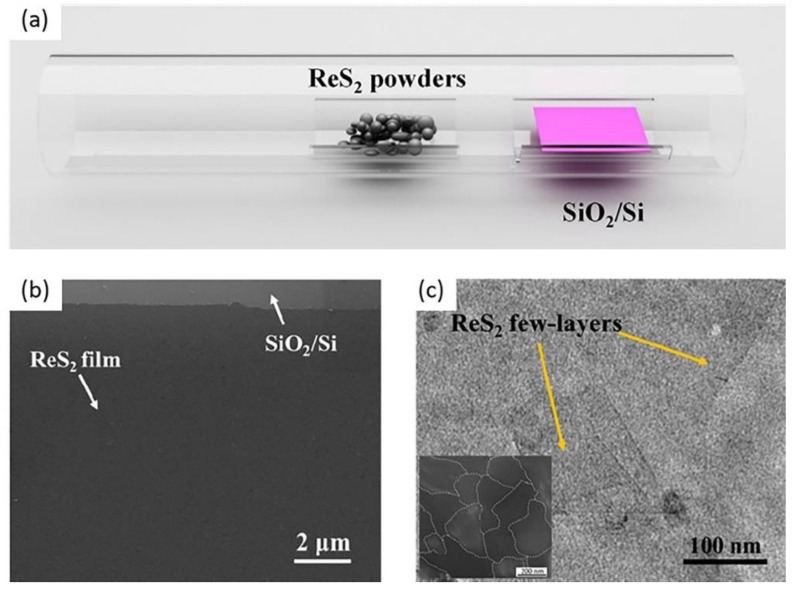
(**a**) Schematic diagram of synthesized ReS_2_ film by PVD. (**b**) SEM image of ReS_2_ film. (**c**) TEM image of the ReS_2_ film. Reprinted with permission from ref. [96]. Copyright 2016 Elsevier.

Physical vapor deposition method is a controlled environmental growth process. The liquid or solid precursors are evaporated in the form of molecules, or atoms in the presence of a low gas pressure within the high vacuum environment to the targeted substrate [94,95]. Synthesized high quality and large area ReS_2_ thin film on top of SiO_2_/Si substrate is obtained by using ReS_2_ powder (99% pure by Alfa-Aesar) with a cost effective, controlled and simple PVD method as reported by Qi et al. [96]. Before pumping the 1-inch quartz tube to vacuum, ReS_2_ powder is placed in the middle of the tube and then filled with argon (Ar) gas as depicted in Figure 8a. The temperature of the furnace is set to 900 °C. After one hour of ReS_2_ growth, the furnace is left to naturally cool down. The AFM is used to measure the thickness of ReS_2_, and a homogenous film of 2.30 nm thickness (three monolayers) is reported. Subsequently, the crystalline structure and morphology is studies using TEM and SEM, respectively. From the SEM image presented in Figure 8b, the surface of ReS_2_ film is found in micrometer size with continuous and clean surface. The as-grown ReS_2_ film is observed in nanometer size from the TEM image presented in Figure 8c. The average grain size is reported to be approximately 250 nm and dark-field TEM (DF-TEM) image is revealed in the inset of Figure 8c. However, higher melting point of the precursor and higher vacuum conditions may be the demerits of this technique for possible adaptability [52].

#### 2.2.4. Chemical Vapor Transport (CVT)

**Figure 9:**
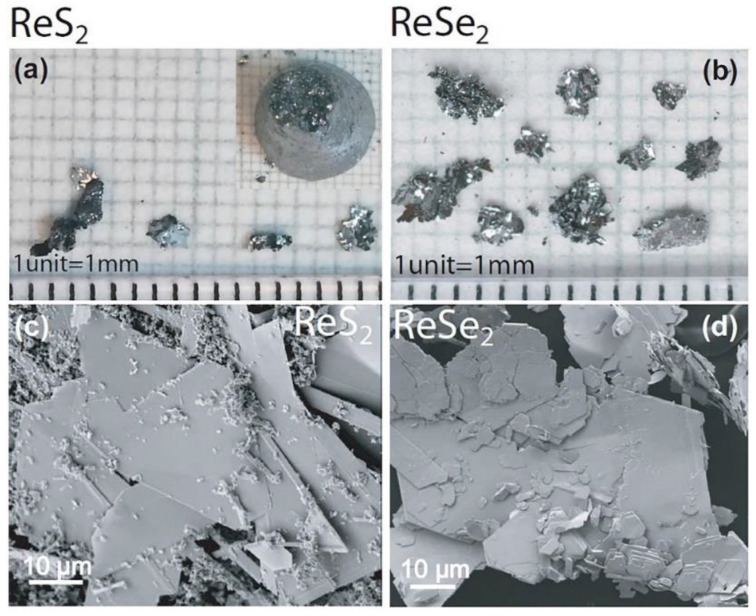
Optical image of the as-grown crystals of (**a**) ReS_2_ (inset shows the ingot as removed from the quartz tube) and (**b**) ReSe_2_. SEM images of (**c**) ReS_2_ and (**d**) ReSe_2_, showing the surface morphology of the flakes. Reprinted with permission from ref. [98]. Copyright 2016 American Chemical Society.

Another highly popular approach for synthesis of bulk or single crystals of ReS_2_ and ReSe_2_ is the chemical vapor transport (CVT) technique. In this method, a sealed ampoule tube is used as the growth chamber, the precursor material and transport agent are placed inside the tube for several to 10 days under low pressure and high temperature [97]. The halogen (I_2_ or Br_2_) is used as the transport agent to aid the growth of ReS_2_ crystals [73]. Nevertheless, this causes the involuntary background doping and the properties of ReS_2_ crystals are also changed [89]. A study reported by Bhakti et al. [98] synthesized high quality ReS_2_ and ReSe_2_ crystals by employing the pure Re and S/Se powders without using halogen transport agent. The growth took place in a cleaned quartz tube at an appropriate temperature using Re and S elements. The shiny plate-like crystals with 20–100 microns thickness is witnessed by optical and SEM image as revealed in Figure 9.

**Figure 10:**
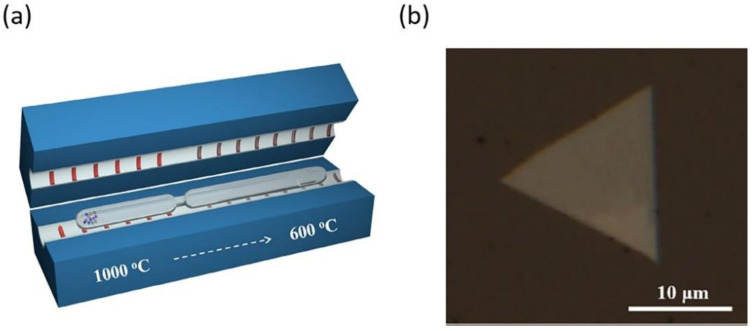
(**a**) A Schematic for the set-up of CVT growth of ReSe2 flakes. The end of ampoule with the source materials and transport agents is placed at the high temperature zone and the other end is placed at the low temperature zone. (**b**) Optical image of a typical CVT-grown ReSe2 flake on mica substrate. Reprinted under the terms of a Creative Commons Attribution 4.0 International License from ref. [99]. Copyright 2019 John Wiley and Sons.

Lei Xing et al. has recently developed a new approach by meticulously tuning the growth kinetics for direct synthesizing of thin ReSe_2_ flakes. The quartz ampoule is specially designed with a neck to separate the powders from the targeted substrate as shown in Figure 10a. This modified method resulted in high quality mono and few layers of ReSe_2_ nanosheets growth on sapphire or mica substrate as shown in Figure 10b [99].

#### 2.2.5. Chemical Vapor Deposition (CVD)

Chemical vapor deposition technique is intensively employed due to high quality, large area and uniform films obtained [100,101]. Large-area monolayer ReS_2_ thin films are exhibited by Keyshar et al. [102] recently using scalable CVD synthesis method. Remarkably, low temperature (450 °C) growth is reported for monolayer synthesis compared to previous studies. The uniform polycrystalline bilayer ReS_2_ film is obtained by synthesizing hexagonal single crystal flakes of ReS_2_ for the first time exploiting CVD method is reported by Hafeez et al. [103]. Three horizontal zones were formed inside the quartz tube within the furnace during the heating process to obtain single and bilayer crystals from the source materials, i.e., ReO_3_ and sulfur. In another study by Dathbun et al. [104], wafer scale uniform ReS_2_ multilayer film is prepared using ReO_3_ and H_2_S gas as precursor. During the infusion of gas, H_2_S directly reacted with ReO_3_ and formed ReS_2_ film of few cm^2^. One of the merits of using this technique is the control on obtained film thickness by regulating the gas flow rate. However, this technology involves harmful and complex transfer procedures from the developed substrate to the laser integration platform in order to construct the SA device, making it difficult to create cost-effective devices [105].

## 3. Photonic Applications

### 3.1. Saturable Absorbers

**Figure 11:**
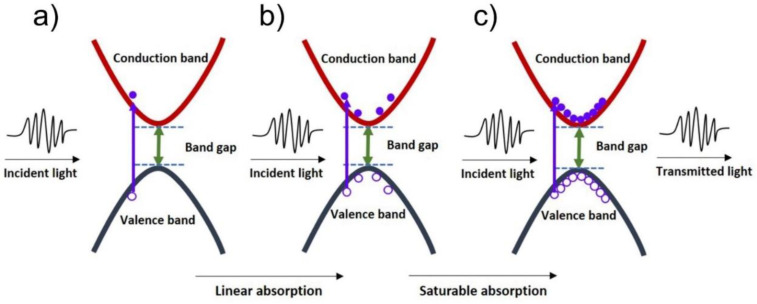
The process of linear absorption and saturable absorption of 2D materials due to Pauli Blocking effect. (**a**) Electrons from the valence band absorb the incident light and are excited to the conduction band. (**b**) More electrons are excited, later they got thermalized and cooled down by the intraband scattering effect. (**c**) When the incident light is sufficiently high, the conduction band is saturated, and the vast majority of light is transmitted rather than absorbed due to the Pauli blocking effect.

The absorption basis of 2D layered materials is primarily the Pauli blocking impact [35,106]; this is schematically depicted in Figure 11. When light having photon energy magnitude more than an incident material’s bandgap falls on the 2D material surface, valence band electrons are excited to the conduction band due to incident photon absorption (Figure 11a). The hot electrons created are swiftly thermalized to institute a hot Fermi–Dirac dissemination. The thermalized carriers are then cooled down more through the intraband scattering impact (Figure 11b). Next, electron-hole recombination process is active until hole distribution is in equilibrium with electron relaxation. Hence, low transmission is the consequence of a majority of incident photons being absorbed. This phenomenon can be attributed for linear optical photon absorption during relatively weak excitation scenario. During high-intensity incident light conditions, there is a substantial increase in photogenerated carriers; consequently, conduction band states are filled with photon energy up to half of their level. This will impede additional absorption due to the Pauli blocking impact (Figure 11c). Pauli blocking effect indicates that two similar electrons cannot occupy an identical state; consequently, light absorption bleaching occurs (majority of the incident light is not absorbed, causing a high transmission) [35].

**Figure 12:**
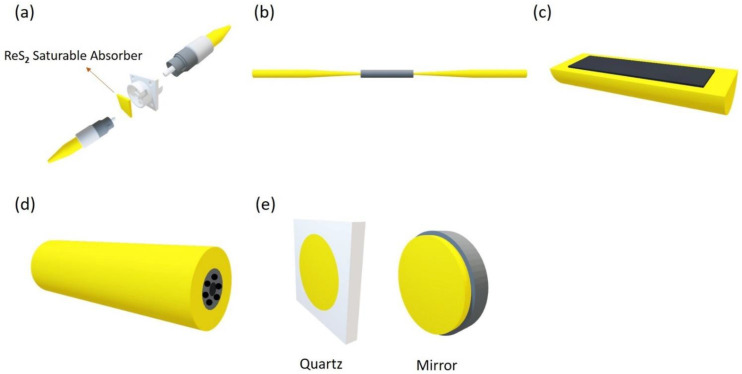
Integration platform for 2D materials to make SA devices. (**a**) Sandwiched between two fiber connecters using fiber adapter. (**b**) Tapered fiber. (**c**) D-shaped (side-polished) fiber. (**d**) Photonic crystal fiber. (**e**) Substrate (e.g., quartz glass plate or mirrors) for free-space coupling.

The produced 2D materials are in the form of thin small sheets having nanometer-scale thickness; hence, they cannot be used directly for lasers. Therefore, it is essential to couple materials into proper optical structures to ease the interface between the light and materials. This type of photonic device having 2D materials utilized in optical fiber or free space structures is known as saturable absorbers (SAs) [58,106]. There are many coupling methods which are summarized in Figure 12. Techniques used for fiber laser applications require material transfer to an end facet of a fiber connector (Figure 12a), tapered fiber (Figure 12b), side-polished fiber (Figure 12c) or filled into the empty photonic crystal fiber (PCF) channels (Figure 12d). It is straightforward and flexible to handle the fiber connector method. A fiber adapter is used to integrate two fibers by placing the SA material in the middle of two end facets. Nevertheless, the obtained material has less damage threshold because SA chemical bonds break due to heat collection due to the high-intensity laser obtained by strong transmission coupling [107]. Side-polished and tapered fiber forms provide the benefit of increased power endurance and damage limits since only a fraction of the light (i.e., evanescent field) interact with the 2D material. Nevertheless, considering that the material has relatively less light intensity, enhancing interaction length can lead to higher non-linearity [108,109]. The PCF scheme is also associated with adequate material-light interaction length and high power-handling ability. In contrast, it is challenging to produce; also, single mode fibers and PCFs have less coupling effectiveness [107]. In the case of solid-state lasers, the coupling schemes are such that there is direct light interaction with the material spin coated on substrates such as mirror or quartz glass surface in free-space using reflection or transmission (Figure 12e).

**Figure 13:**
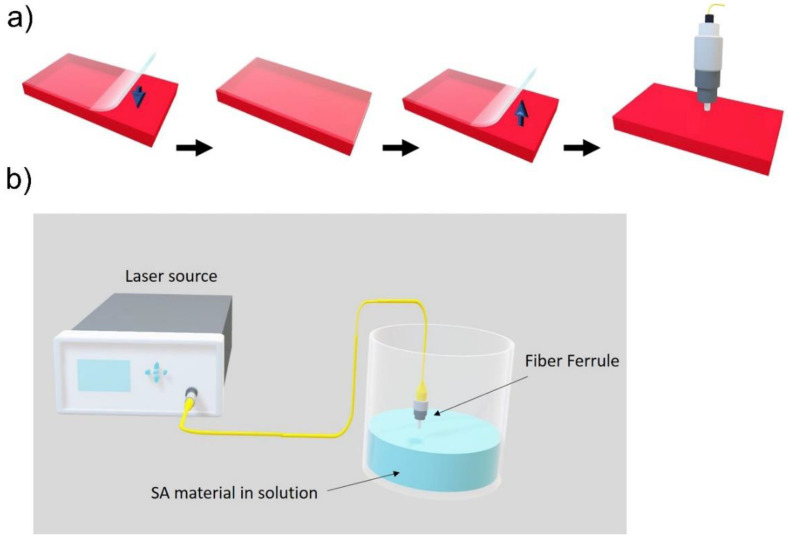
(**a**) Transferring a mechanically exfoliated few-layer flakes to fiber connecter. (**b**) Deposition of SA material onto the optical fiber ferrule based on optical driven deposition technique.

Choosing an appropriate technique for transferring material onto optical devices relies on material production techniques. Considering the mechanical exfoliation method, it is possible to move flake layers to the fiber connector by pressing down the end facet on scotch tape having few-layer thick peeled flakes. Consequently, the adhesive force between the ceramic and flakes causes a 2D-material layer to deposit on the fiber core (Figure 13a) [41]. Optical-driven deposition may be employed for solution-based exfoliation. Initially, the fiber’s end facet is dipped inside the solution; subsequently, a strong beam of light is injected. Consequently, the fiber tip becomes coated with the material because of temperature-gradient induced material movement [110,111,112], as depicted in Figure 13b. Additionally, solution exfoliation nanomaterials can now be transferred using inkjet printing technology. This technique comprises an ink based on 2D flakes; the ink is deposited on the substrate surface, thereby facilitating precisely controlled production at scale [113,114]. Moreover, these nanomaterials may be spread using polymer films such as PMMA, PVA or other substances. Subsequently, the composite layer is placed in the middle of fiber connectors [115].

### 3.2. Nonlinear Absorption Characterization

As per the nonlinear optical theory [116], the expression of the absorption of sample could be acquired based on the relation between the absorption coefficient α and intensity of incident light I as given in Equation (1).

(1) $$\alpha \left(I\right)=\alpha_{0}+{\;\alpha }_{\mathrm{NL}\;}I$$

here, $\alpha_{0}$ denotes the linear absorption coefficient and $\alpha_{\mathrm{NL}\;}$ represents the nonlinear absorption coefficient. The expression of nonlinear absorption of SAs could be acquired as shown in Equation (2) [35,117,118].

(2) $$\alpha \left(I\right)=\frac{\alpha_{s}}{1+\frac{I}{I_{\mathrm{sat}}}}+\alpha_{\mathrm{ns}}$$

here, $\alpha_{s}$ denotes the saturable loss (also called as modulation depth ΔR or ΔT), $I_{\mathrm{sat}}$ signifies the saturation intensity and $\alpha_{\mathrm{ns}}$ represents the non-saturable loss. Other key parameters pertaining to SAs include the wavelength range (where it absorbs) as well as recovery time $\tau_{A}$, which need to be of very short time in order to enable passive mode-locking, but not too short with regards to passive Q-switching [119].

**Figure 14:**
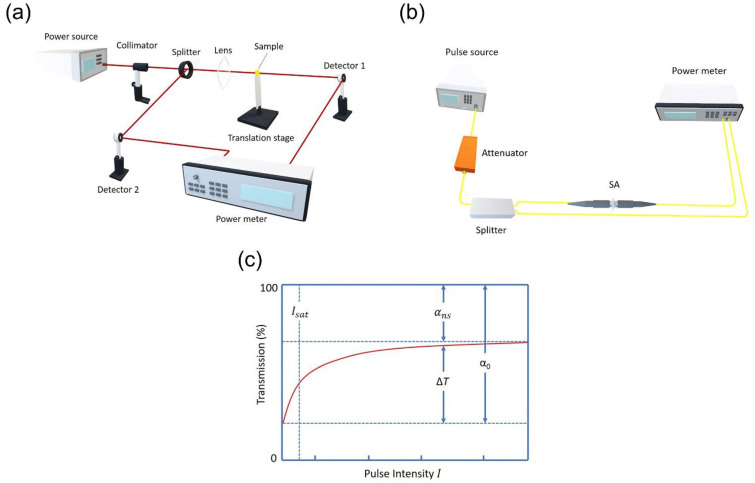
(**a**) Z-scan measurement (**b**) I-scan measurement (or balanced twin-detector technique) (**c**) Example of a transmission curve.

For convenience, expression of I can be acquired as the energy E as well as the incident light’s fluence F, thus $I_{\mathrm{sat}}$ in Equation (2) could be replaced by the saturation energy $E_{\mathrm{sat}}$ as well as saturation fluence $F_{\mathrm{sat}}$ [120]. Various SA parameters with their units and definitions are summarized in Table 1 below:

Two common measurement techniques can be employed to characterize these SA parameters: I-scan measurement (often called as the balanced twin-detector technique) [121] and Z-scan measurement [116,122]. The Z-scan measurement technique allows characterizing the free-space-type SA sample. As presented in Figure 14a, with the help of a splitter, the pulsed light is first split into two beams of light from the pumping source. The measurement beam would be the beam that propagates along the SA sample’s incorporated path, while the other beam would be regarded as the reference beam. The measurement beam is focused by a lens to the SA sample, which has been mounted on a Z-direction translation stage. Changing of light intensity per unit area of the sample can be acquired based on various beam sizes by moving the sample towards the z-axis along with the measurement beam. The dual-channel power meter is employed to collect the power from both paths. The power from the two detectors is compared in order to obtain the sample’s nonlinear absorption. Closed-aperture Z-scan measurement may explore the Kerr effect of the sample and acquire the nonlinear index because the beam diameter after the sample is connected to the beam induced refractive index change of the sample [116]. Balanced twin-detector measurement is chosen for SAs that have been integrated as the fiber-based device to perform nonlinear absorption characterization. The principle can be said to be analogous to Z-scan, except that the setup is completely fiberized (Figure 14b). An optical coupler is employed to split the light from a pulsed laser into reference and measurement beams. In contrast with the Z-scan method, a variable optical attenuator is mounted before the optical coupler in order to achieve the variation in light intensity for twin-detector measurement [58].

The optical attenuator is employed to change the input power gradually, which enables recording a series of optical transmittance based on different input intensities. Then, characterization and plotting (Figure 14c) of the corresponding nonlinear optical parameters can be acquired via fitting the relation between the input laser power (I) and the optical transmission rate T(I) based on Equation (3) [108].

(3) $$T\left(I\right)=1-\Delta T*\exp\left(-I/I_{\mathrm{sat}}\right)-{\;\alpha }_{\mathrm{ns}}$$

Based on the operation of Q-switching or mode-locking, integrating SA devices within the laser cavity can aid in creating short optical pulses. Usually, Q-switching can generate pulses possessing high energy (μJ–mJ) at low repetition frequency (kHz) as well as pulse durations in the range of μs–ns. While shorter durations (ps–fs) at higher repetition rate (MHz–GHz) are associated with the mode-locked laser pulses, they also possess lower energy versus Q-switched lasers (pJ–μJ). Both mode-locking and Q-switching can exist together within the same laser cavity but with differing thresholds. Various factors such as gain and loss in cavity due to alteration of input power, and balancing between cavity’s dispersion and nonlinearity [114] can impact the transition between the two operations.

### 3.3. Q-Switched Lasers

Typically, Q-switching operation gives pulses possessing higher energy as well as peak power versus mode-locked lasers. Table 2 presents the performance pertaining to Q-switched fiber lasers as well as solid-state lasers with regards to ReS_2_-SA. X. Su et al. presented the initial work [62], in which they showed a Q-switched Er:YSGG solid-state pulsed laser at 2.8 μm along with 324 ns pulse duration. Later, various bulk gain medium such as Er:SrF2, Pr:YLF, Nd:GdLaNbO4, Tm:YAP and Nd:YAG, were employed, which encompassed broad wavelengths ranging from 640 nm to 2950 nm [68,123]. The ReS_2_ was spin-coated on various substrates such as mirror, quartz, sapphire, Yttrium, aluminum garnet (YAG) and K9 glass in order to fabricate the SA device [71,124,125].

With regards to fiber-based laser system, Xu et al. and B. Lu et al. employed Erbium and Ytterbium-doped fibers in order to produce Q-switched laser pulses at 1 μm and 1.5 μm waveband with an average output power of 2.48 mW and 3.2 mW [61,126], respectively. In contrast to fiber lasers, solid-state pulsed lasers offer the benefits of low undesirable nonlinear impacts as well as a wider mode area, which make them optimum for applications that need high power sources [35]. Q-switched solid-state laser possessing maximum average output power of 580 mW as well as highest peak power of 22.1 W was reported by M. Fan et al. who employed Er:SrF_2_ crystal as gain medium [125].

**Figure 15:**
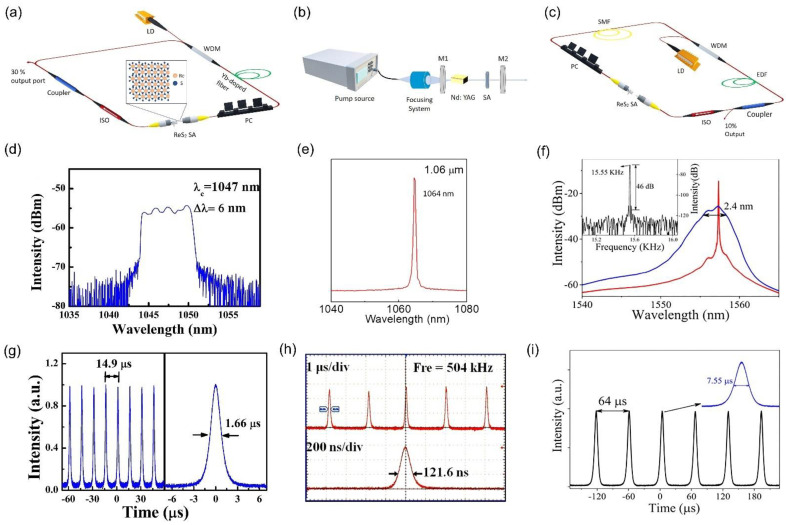
Q-switched pulsed lasers with ReS_2_ SA. (**a**,**c**) cavity designs; (**d**,**f**) output spectrum; (**g**,**i**) output pulse trains and the corresponding pulse profile along with the repetition rate and pulse duration. (**a**) Adapted with permission from ref. [126]. Copyright 2018 IEEE. (**b**) Adapted with permission from ref. [69]. Copyright 2019 John Wiley and Sons. (**c**) Adapted with permission from ref. [63]. Copyright 2018 IEEE. (**d**,**g**), Reprinted with permission from ref. [126]. Copyright 2018 IEEE. (**e**,**h**), Reprinted with permission from ref. [69]. Copyright 2019 John Wiley and Sons. (**f**,**i**), Reprinted with permission from ref. [63]. Copyright 2018 IEEE.

Apart from the wide application in infrared lasers, ReS_2_-SA is employed for the visible spectrum region. X. Su et al. designed a passively Q-switched laser at 640 nm, which possessed 160 ns pulse duration and 52 mW average output power [123]. Moreover, a Q-switched Nd-doped crystal laser was reported by Han et al. close to ReS_2_ bandgap at 950 nm [71]. Various schematic setups are demonstrated in Figure 15 in order to generate Q-switched lasers based on ReS_2_-SA as well as their output characteristics at 1047 nm, 1064 nm and 1557.3 nm [63,69,126].

### 3.4. Mode-Locked Lasers

**Figure 16:**
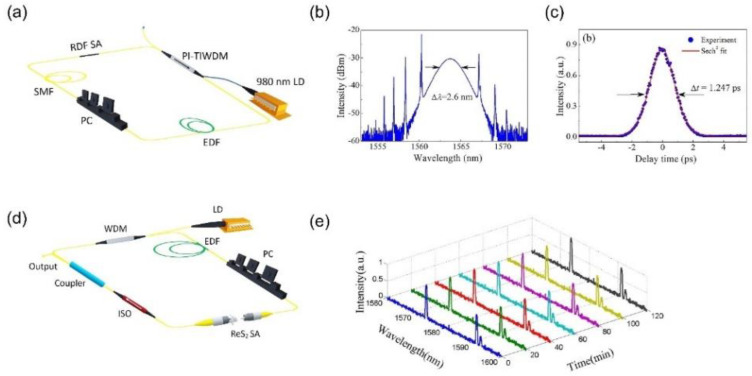
Mode-locked laser pulses at 1.5 μm. (**a**) Laser setup using ReS_2_-covered D-shaped fiber SA (RDF SA). (**b**) Output optical spectrum with a spectral width Δλ of 2.6 nm. (**c**) Autocorrelation trace of the experimental data (dots) and Sech^2^-shaped fit (solid curve) (**d**) Laser setup with ReS_2_ sandwiched between two fiber connectors to form the SA. (**e**) The corresponding multi-wavelength output with long-term stability over 2 h. (**a**) Adapted under the terms of a Creative Commons Attribution 4.0 International License from ref. [60]. Copyright 2017 Springer Nature. (**b**,**c**), Reprinted under the terms of a Creative Commons Attribution 4.0 International License from ref. [60]. Copyright 2017 Springer Nature. (**d**) Adapted under the terms of a Creative Commons Attribution 4.0 International License from ref. [65]. Copyright 2018 The Optical Society. (**e**) Reprinted under the terms of a Creative Commons Attribution 4.0 International License from ref. [65]. Copyright 2018 The Optical Society.

The performance of mode-locked lasers based on ReS_2_ SA is shown in Table 3. As observed, majority of the reports were found to be on near-infrared communication band at 1.5 μm by employing Erbium fiber as gain medium. High quality beam can be produced via fiber lasers platforms along with alignment-free, compact and low-cost structure [30]. In 2017, first identification of mode-locking operation that was based on ReS_2_-SA was acquired [60]. By including the D-shaped fiber possessing ReS_2_–polymer composite, generation of mode-locked pulses centered at 1564 nm with pulse duration of 1.25 ps was acquired as demonstrated in Figure 16a–c. In addition, stable mode-locked pulses at 1563.3 nm possessing pulse width of 3.8 ps was reported by Xu et al. The nature of double covered ReS_2_ microfiber structure allowed obtaining high optical damage threshold of 410 mw [67]. In addition, a multi-wavelength Er-doped fiber laser (1573.5 nm, 1591.1 nm and 1592.6 nm) was produced by sandwiching ReS_2_ between the two fiber connecters as presented in Figure 16d [65]. Figure 16e displays the output spectrum, which was monitored for about 2 h to observe long-term stability operation. Similarly, Mao et al. employed a fiber connecter to build a ReS_2_-SA device inside the Erbium-doped fiber laser (EDFL) system, which allowed obtaining self-started mode-locked pulses that were centered at 1558.6 nm along with the duration of 1.6 ps by altering the polarization controller [63]. The first report was realized with regarding mode-locked laser employing ReS_2_ in longer wavelengths at 2 μm waveband in thulium-doped fiber laser possessing 893 fs pulse duration as well as 4.13 mw maximum average power [128]. Su et al. employed Yb:CALGO solid-state laser as well as ReS_2_ saturable absorption mirror (SAM) to produce 1060 nm mode-locked laser pulses with higher average output power of 350 mW as well as shorter pulse duration of 323 fs [123]. These reports demonstrate that ReS_2_ can be employed as broadband SA for various wavelengths, at discrete or multiwavelength operation.

Apart from the broadband saturable absorption, we can also employ large third-order nonlinear effects of ReS_2_ to yield pulses possessing high repetition rates with potential applications in frequency combs and soliton communications. Feifei Lu produced pulses with repetition rate, which could reach as high as 318.5 MHz and corresponded to the 168th harmonic mode-locking of Erbium-doped fiber laser [17].

## 4. Discussion and Outlook

**Figure 17:**
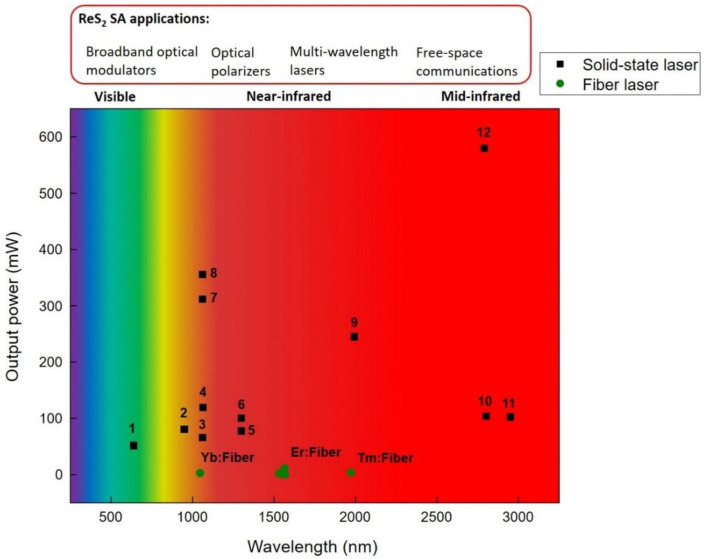
Spectral coverage for several types of pulsed fiber lasers and solid-state lasers generated by ReS_2_ SA along with the corresponding output power. 1, Pr:YLF crystal [123]: 2, Nd:YAG crystal [71]: 3, Nd:YAG crystal [69]: 4, Nd:YAG crystal [123]: 5, Nd:YAG crystal [70]: 6, Nd:YAG crystal [69]: 7, Nd:GdLaNbO_4_ crystal [127]: 8, Nd:YSAG crystal [124]: 9, Tm:YAP crystal [123]: 10, Er:YSGG crystal [62]: 11, Ho,Pr:LLF crystal [68]: 12, Er:SrF2 crystal [125].

As for the reported results on employing ReS_2_ as saturable absorber, the laser performance showed steady improvements. With regards to integration, variety of transfer methods were utilized, such as embedding ReS_2_ within polymer composite [64], optical driven deposition [65], spin coating [123] and drop casting [69]. Material fabrication techniques exhibited consistent progress in being more controllable such as CVD [127] and low-cost fabrication such as mechanical exfoliation [126]. As depicted in Figure 17, the operation bands of ReS_2_-based SA have been employed successfully for various wavelength regions ranging from visible to mid-infrared (0.64–3 μm) in fiber-based as well as solid state laser systems [61,68,71,123,128] leading to several optical applications.

The bandgap of ReS_2_ amounts to ~1.4 eV which corresponds to an optical absorption at ~800 nm wavelength [130]. Nonetheless, previous works have reported generation of Q-switched and mode-locked lasers using ReS_2_ functioning as SA at 1550 nm (~0.8 eV) and 2950 nm (~0.4 eV), which is much less than the bandgap of ReS_2_. Thus, in theory, there ought to be no light absorption. Nonetheless, the absorption occurs due to the unavoidable deficiencies in the formation of the material, e.g., grain boundaries and point defects [60]. The introduction of crystallographic or the edge state defect in the material enables the absorption of light photons with energies less than the material bandgap (i.e., sub-bandgap absorption) [131]. By leveraging the effect of sub-bandgap absorption, ReS_2_ based SA could consequently support pulsed lasers in the mid-infrared and near-infrared wavelengths where energies of the photons are less than the intended material bandgap. Various theories have been developed to rationalize the sub-bandgap absorption of TMDs, namely decrease in bandgap due to defects [132,133], materials’ edge state [134] (can be considered as a special defect at the nanosheets’ edges) and saturation of two-photon absorption [135]. X. Su et al. computed the ReS_2_ band structure with various ratios of Re and S by using technique of Vienna ab initio simulation, and the results suggest that the bandgap can be decreased from 1.38 to 0.56 eV by introducing S defects in an appropriate range [123]. Moreover, Horzum et al. theoretically examined the atomic imperfections in monolayer ReS_2_. It was observed that the S vacancy formation decreases the bandgap from 1.43 to 1.08 eV and the bandgap with Re imperfections becomes only 0.35 eV [136].

The modulation depth, as the main classification parameter of the nonlinear absorption of SA, which is significantly influenced by the material thickness, has a considerable impact on the laser performance. Considerable modulation depth is advantageous for producing short pulses. A concern for monolayer ReS_2_-SA (thickness of 0.7 nm [54]) is the low modulation depth that is generally 1% only [60]. By increasing the ReS_2_ thickness, the modulation depth can be increased. B. Lu et al. attained up to 44% modulation depth by piling up 30 layers (21 nm) of ReS_2_ [126], though this also increases the thresholds of saturation intensity. This is due to the fact that thicker films have higher density of localized defective states on the grain edge and more photons are trapped [67,137]. Intensity/fluence of high saturation implies that the pulsed function can be initiated at higher power level.

Nonetheless, increasing the thickness may have a reverse or positive impact on modulation depth [107]. Mao et al. obtained only 0.12% modulation depth with 6 layers (4 nm) of ReS_2_ [63]. ReS_2_-SA performance also depends on the wavelength of operation. X. Su et al. demonstrates a reduction in the saturation fluence at higher wavelengths, for instance, 58.2 μJ/${\mathrm{cm}}^{2}$ at 640 nm, 21.5 μJ/${\mathrm{cm}}^{2}$ at 1064 nm and 2.7 μJ/${\mathrm{cm}}^{2}$ at 1991 nm [123]. This dependence on wavelength makes the use of ReS_2_-SA in the mid-infrared region more favorable.

Saturable absorbers having low optical damage threshold can lead to collapse of pulsed operation and instead of that, the laser engages in CW operation, particularly when the material becomes deposited on the fiber connector’s tip, where the laser connects directly with the material. For instance, pump power above 330 mW caused strong signal jittering to dominate and obliterate the Q-switched pulses [61]; nonetheless, the Q-switched pulses could be obtained again just by reducing the pump power. This is because of the over-saturation of ReS_2_-SA instead of thermal damage [138,139]. Many attempts have been carried out to increase the ReS_2_-SA damage threshold by utilizing the evanescent field effect through D-shaped or tapered fibers. X. Xu et al. attained stable mode-locking pulses with comparatively high laser damage threshold of 410 mW through double covered ReS_2_ micro-fiber as SA device [67].

One of the challenges lies in a proper deposition/transferring process of the material into the laser system. If the material is not distributed entirely and evenly on the laser integration platform, only a fraction of the region of ReS_2_ can sustain the Q-switching/mod-locking operation which may cause deterioration in long-term stability performance. Moreover, ReS_2_ production is costly due to its complex structure and low abundance on earth [140]. Furthermore, generating finely tunable pulsed laser with high performance based on ReS_2_-SA is still difficult. Using ultrafast laser in the mid-infrared region can advance the chemical and biomedical sensing applications, since many absorption bands of molecular solids, liquids and gases can be found in this particular region [141]. However, only one study has reported ultrafast sub-picosecond mode-locked pulses in the MIR region using ReS_2_-SA [128].

A future direction for the SAs is to investigate the integration of the emergent ReS_2_ with conventional well-established materials such as MoS_2_ and WS_2_ to form heterostructures [142,143,144,145]. This is likely to help in achieving SAs with greater nonlinearity, higher damage threshold, as well as ultrafast relaxation time by using the properties of various materials. Furthermore, developing hybrid SAs by using artificial SAs such as NPE, and real SAs can increase the parameter modulation depth and improve the performance of the output laser. Several applications will be benefited from such ultrafast lasers, and we suppose ReS_2_ is a potential material for SAs devices based on different laser sources.

Finally, we note that ReS_2_ is only one member of the TMDs family which has unique and strong anisotropic properties. We believe that further study on these properties can stimulate the use of other similar low symmetry 2D materials in developing innovative photonic devices.

## 5. Conclusions

In this review, we have emphasized how the property of broadband absorption and large third-order nonlinear effect of ReS_2_ can help in the development of flexible and low-cost SAs integrated in fiber-based and solid-state lasers. The ReS_2_-SA has been effectively used in short pulse generation, providing pulses with maximum peak power up to 22 W, broadband wavelength extending from visible (640 nm) to mid-infrared (2.95 μm) range, and pulse widths down to as low as 270 fs. Moreover, high signal-to-noise ratio (SNR) of more than 70 dB was observed, which demonstrates the high level of pulse stability. These outcomes show that ReS_2_ is a potential material for several applications such as broadband optical modulator, pulsed lasers and sensors. Majority of the studies utilized liquid-phase exfoliation to form ReS_2_. Liquid-phase exfoliation technique is an efficient, convenient and cheap method to produce 2D materials. Molecular beam epitaxy (MBE) is another interesting fabrication method which has not been employed yet for ReS_2_ synthesis. MBE is regarded as one of the most effective techniques for controlling the number of layers while maintaining monolayer homogeneity. This review produces an introduction to the potential of ReS_2_ as novel saturable absorber for ultrafast photonic technology. Nonetheless, the most challenging concern is to use these controlled laboratory photonic devices in environmentally stable industrial applications.

## Figures and Tables

**Table 1:** SA parameters, their units and definitions.

Parameter	Symbol	Unit	Definition
Saturation intensity	$I_{\mathrm{sat}}$	$W/{\mathrm{cm}}^{2}$	The required intensity/energy/fluence to reduce absorption by 0.5 $\alpha_{s}$
Saturation energy	$E_{\mathrm{sat}}$	J	
Saturation fluence	$F_{\mathrm{sat}}$	J$/{\mathrm{cm}}^{2}$	
Recovery time	$\tau_{A}$	S	The decay time of the excitation after an exciting pulse
Modulation depth (also known as saturable loss $\alpha_{s}$)	ΔT ^1^, ΔR or $\alpha_{s}$	in %	Maximum possible change in optical loss $\alpha_{s}=\alpha_{0}-\alpha_{\mathrm{ns}}$[114]
Non-saturable loss	$\alpha_{\mathrm{ns}}$ ($\Delta T_{\mathrm{ns}}$ or $\Delta R_{\mathrm{ns}}$)	in %	Typically, unwanted part of the losses which cannot be saturated, meaning that the SA device will not reach 100% reflectivity or transmission, even for arbitrarily high pulse intensity [119]

^1^ In case the saturable absorber is used in transmission ΔT or reflection ΔR structure.

**Table 2:** Nonlinear optical characteristics and applications in laser cavities using ReS_2_ for Q-switched lasers.

**Gain** **Medium**	**Fabrication** **Method**	**Integration** **Platform**	**ReS_2_ Thickness**	Nonlinear Characterization		Laser Parameters						Ref
				**Modulation Depth [%]**	Saturation Level	$\mathrm{Wavelength}\;\mathrm{Regime}\;(\lambda_{\mathrm{center}})$	$f_{rep}\;[\mathrm{kHz}]$	$\tau_{p}\;[\mathrm{ns}]$	$P_{peak}\;[W]$	$E_{p}\;[\mathrm{nJ}]$	$P_{out}\;[\mathrm{mW}]$	
Yb:Fiber	ME	Fiber Ferrule	21 nm (30 layers)	44	8.4 MW/${\mathrm{cm}}^{2}$	1 μm (1047 nm)	134	1560	0.00813	13.02	3.2	[126]
Er:Fiber	ME	Fiber Ferrule	-	-	150 GW/${\mathrm{cm}}^{2}$	1.5 μm (1532 nm)	64	2100	-	38	2.48	[61]
Er:Fiber	LPE	Fiber Ferrule	∼4 nm (6 layers)	0.12	74 MW/${\mathrm{cm}}^{2}$	1.5 μm (1557.3 nm)	19	5496	-	62,800	1.2	[63]
Er:Fiber	LPE	Fiber Ferrule	5 nm (7 layers)	-	-	1.5 μm (1550 nm)	66.52	2400	-	18.88	1.25	[64]
Pr:YLF Nd:YAG Tm:YAP crystals	LPE	Mirror	4 nm (6 layers)	3 5.2 2.9	58.2 μJ/${\mathrm{cm}}^{2}$ 21.5 μJ/${\mathrm{cm}}^{2}$ 2.7 μJ/${\mathrm{cm}}^{2}$	Visible (640 nm) 1 μm (1064 nm) 2 μm (1991 nm)	520 644 677	160 139 415	0.625 1.34 8.72	-	52 120 245	[123]
Nd:YAG crystal	LPE	Quartz	15 layers	0.33	2.54 GW/${\mathrm{cm}}^{2}$	Visible (946 nm) 1 μm (1064 nm)	165	834	-	491	81	[71]
Nd:GdLaNbO_4_ crystal	CVD	Sapphire	3.5–5 nm (5–7 layers)	-	-	1 μm (1060 nm)	147	400	5.3	2120	312	[127]
Nd:YSAG crystal	LPS	K9 glass	6 nm	7.4	207.19 MW/${\mathrm{cm}}^{2}$	1 μm (1060 nm)	70	390	-	-	356	[124]
Nd:YAG crystal	LPE	Sapphire	1.4–2 nm (2–3 layers)	2 4	2.17 KW/${\mathrm{cm}}^{2}$ 0.55 KW/${\mathrm{cm}}^{2}$	1 μm (1060 nm, 1300 nm)	504 308.4	121.6 111	1.08 2.95	130 330	66 101	[69]
Nd:YAG crystal	LPE	Sapphire	4 nm (6 layers)	15	15.6 μJ/${\mathrm{cm}}^{2}$	1 μm (1300 nm)	214	403	0.9	420	78	[70]
Er:YSGG crystal	LPE	Sapphire	3.5 nm (5 layers)	9.7	22.6 μJ/${\mathrm{cm}}^{2}$	3 μm (2800 nm)	126	324	2.56	825	104	[62]
Er:SrF2 crystal	LPE	Yttrium aluminum garnet (YAG)	2.2 to 7.2 nm (3–10 layers)	3.8	-	3 μm (2790 nm)	49	508	22.1	12,100	580	[125]
Ho,Pr:LLF crystal	LPE	Sapphire	4 nm (6 layers)	10.2	23.5 μJ/${\mathrm{cm}}^{2}$	3 μm (2950 nm)	91.5	676	1.67	1130	103	[68]

ME, mechanical exfoliation; LPE, liquid-phase exfoliation; CVD, chemical vapor deposition; LPS, liquid-phase stripping; $f_{\mathrm{rep}}$, repetition frequency; $\tau_{p}$, pulse duration; $P_{\mathrm{peak}}$, pulse peak power; $E_{p}$, pulse energy; $P_{\mathrm{out}}$, output power.

**Table 3:** Nonlinear optical characteristics and applications in laser cavities using ReS_2_ for mode-locked lasers.

**Gain** **Medium**	Fabrication Method	Integration Platform	Nonlinear Characterization		Laser Parameters						Ref
			Modulation Depth [%]	Saturation Level	$\mathrm{Wavelength}\;\mathrm{Regime}\;(\lambda_{center})$	Δλ[nm]	$f_{rep}\;[\mathrm{MHz}]$	$\tau_{p}\;[\mathrm{fs}]$	$P_{out}\;[\mathrm{mW}]$	SNR [dB]	
Yb:CALGO crystal	LPE	Mirror	5.2	21.5 μJ/${\mathrm{cm}}^{2}$	1 μm (1060 nm)	4.23	50.7	323	350	60	[123]
Er:Fiber	CVD	D-shaped fiber	1	27 μJ/${\mathrm{cm}}^{2}$	1.5 μm (1564 nm)	2.6	3.43	1250	-	60	[60]
Er:Fiber	CVD	Microfiber	-	-	1.5 μm (1564.4 nm)	0.45	1.896 (318.5 for HML)	2549	12	40	[17]
Er:Fiber	LPE	Fiber Ferrule	6.9	27.5 μJ/${\mathrm{cm}}^{2}$	1.5 μm (1573.6 nm, 1591.1 nm, 1592.6 nm)	17.5	13.39	-	-	55	[65]
Er:Fiber	LPE	Fiber Ferrule	0.12	74 MW/${\mathrm{cm}}^{2}$	1.5 μm (1558.6 nm)	1.85	5.48	1600	0.4	-	[63]
Er:Fiber	LPE	Double-Covered Microfiber	0.25	410 mW	1.5 μm (1563.3 nm)	8.2	1.78	3800	-	68	[67]
Er:Fiber	ME	D-shaped fiber	-	-	1.5 μm (~1560 nm)	15.4	14.53	270	1.08	-	[129]
Er:Fiber	LPE	Microfiber	-	-	1.5 μm (1565 nm)	10.7	20	-	40	80	[66]
Tm:Fiber	Hydrothermal synthesis	Side-polished fiber	62	73.6 W	2 μm (1970.65 nm)	5.05	26.1	893	4.13	70	[128]

Δλ, spectral bandwidth; HML, harmonic mode-locking.

## References

[1] Keller U. (2003). Recent developments in compact ultrafast lasers. Nature.

[2] Shi W., Fang Q., Zhu X., Norwood R.A., Peyghambarian N. (2014). Fiber lasers and their applications. Appl. Opt..

[3] Kobayashi T. (2018). Development of ultrashort pulse lasers for ultrafast spectroscopy. Photonics.

[4] Morgenweg J., Barmes I., Eikema K.S.E. (2014). Ramsey-comb spectroscopy with intense ultrashort laser pulses. Nat. Phys..

[5] Udem T., Holzwarth R., Hänsch T.W. (2002). Optical frequency metrology. Nature.

[6] Muhanad Fadhel M., Rashid H., Essa Hamzah A., Dzulkefly Zan M.S., Abd Aziz N., Arsad N. (2021). Flat frequency comb generation employing cascaded single-drive Mach–Zehnder modulators with a simple analogue driving signal. J. Mod. Opt..

[7] Elgaud M.M., Bakar A.A.A., Ghaith A.A., Naim N.F., Arsad N., Mokhtar M.H.H., Azeman N.H., Zan M.S.D. (2018). Pulse Compressed Time Domain Multiplexed Fiber Bragg Grating Sensor: A Comparative Study. IEEE Access.

[8] Hamzah A.E., Zan M.S.D., Elgaud M., Fadhel M.M., Alwash S.A., Abushagur A.A., Mokhtar M.H.H., Azeman N.H., Ali S.H.b.M., Bakar A.A.A. Signal Generation using System on Chip for Coded Fiber Bragg Grating Sensor. Proceedings of the 2020 IEEE 8th International Conference on Photonics (ICP).

[9] Zan M.S.D., Elgaud M.M., Zainuddin A.R., Kadhim A.S., Mokhtar M.H.H., Arsad N., Bakar A.A.A. (2021). Simulation Analysis on the Simultaneous Deployment of Brillouin Gain and Loss in Coded Brillouin Optical Time Domain Analysis (BOTDA) Fiber Sensor. J. Phys. Conf. Ser..

[10] Liu X., Cui Y., Han D., Yao X., Sun Z. (2015). Distributed ultrafast fibre laser. Sci. Rep..

[11] Taha B.A., Ali N., Sapiee N.M., Fadhel M.M., Mat Yeh R.M., Bachok N.N., Al Mashhadany Y., Arsad N. (2021). Comprehensive Review Tapered Optical Fiber Configurations for Sensing Application: Trend and Challenges. Biosensors.

[12] Ippen E.P. (1994). Principles of passive mode locking. Appl. Phys. B.

[13] Liu J., Wu J., Chen H., Chen Y., Wang Z., Ma C., Zhang H. (2020). Short-pulsed Raman fiber laser and its dynamics. Sci. China Phys. Mech..

[14] Haus H.A. (2000). Mode-locking of lasers. IEEE J. Sel. Top. Quantum Electron..

[15] Xia W., Chen X. (2016). Recent developments in fiber-based optical frequency comb and its applications. Meas. Sci. Technol..

[16] Woodward R.I., Kelleher E.J. (2015). 2D saturable absorbers for fibre lasers. Appl. Sci..

[17] Lu F. (2017). Passively harmonic mode-locked fiber laser based on ReS_2_ saturable absorber. Mod. Phys. Lett. B.

[18] Soffer B.H. (1964). Giant Pulse Laser Operation by a Passive, Reversibly Bleachable Absorber. J. Appl. Phys..

[19] Bret G., Gires F. (1964). Giant-Pulse Laser and Light Amplifier Using Variable Transmission Coefficient Glasses as Light Switches. Appl. Phys. Lett..

[20] Maiman T.H. (1960). Stimulated Optical Radiation in Ruby. Nature.

[21] Ippen E.P., Shank C.V., Dienes A. (1972). Passive mode locking of the cw dye laser. Appl. Phys. Lett..

[22] Dzhibladze M.I., Ésiashvili Z.G., Teplitskiĭ É.S., Isaev S.K., Sagaradze V.R. (1983). Mode locking in a fiber laser. Sov. J. Quantum Electron..

[23] Keller U., Miller D., Boyd G., Chiu T., Ferguson J., Asom M. (1992). Solid-state low-loss intracavity saturable absorber for Nd: YLF lasers: An antiresonant semiconductor Fabry–Perot saturable absorber. Opt. Lett..

[24] Bogusławski J., Wang Y., Xue H., Yang X., Mao D., Gan X., Ren Z., Zhao J., Dai Q., Soboń G. (2018). Graphene Actively Mode-Locked Lasers. Adv. Funct. Mater..

[25] Wen Q.-Y., Tian W., Mao Q., Chen Z., Liu W.-W., Yang Q.-H., Sanderson M., Zhang H.-W. (2014). Graphene based All-Optical Spatial Terahertz Modulator. Sci. Rep..

[26] Zhang H., Healy N., Shen L., Huang C.C., Hewak D.W., Peacock A.C. (2016). Enhanced all-optical modulation in a graphene-coated fibre with low insertion loss. Sci. Rep..

[27] Cho B., Hahm M.G., Choi M., Yoon J., Kim A.R., Lee Y.-J., Park S.-G., Kwon J.-D., Kim C.S., Song M. (2015). Charge-transfer-based Gas Sensing Using Atomic-layer MoS_2_. Sci. Rep..

[28] Wang S., Wang J., Zhao W., Giustiniano F., Chu L., Verzhbitskiy I., Zhou Yong J., Eda G. (2017). Efficient Carrier-to-Exciton Conversion in Field Emission Tunnel Diodes Based on MIS-Type van der Waals Heterostack. Nano Lett..

[29] Sun X., Qiu C., Wu J., Zhou H., Pan T., Mao J., Yin X., Liu R., Gao W., Fang Z. (2015). Broadband photodetection in a microfiber-graphene device. Opt. Express.

[30] Martinez A., Sun Z. (2013). Nanotube and graphene saturable absorbers for fibre lasers. Nat. Photonics.

[31] Hu X., Yasaei P., Jokisaari J., Öğüt S., Salehi-Khojin A., Klie R.F. (2018). Mapping thermal expansion coefficients in freestanding 2D materials at the nanometer scale. Phys. Rev. Lett..

[32] Guerreiro P.T., Ten S., Borrelli N.F., Butty J., Jabbour G.E., Peyghambarian N. (1997). PbS quantum-dot doped glasses as saturable absorbers for mode locking of a Cr:forsterite laser. Appl. Phys. Lett..

[33] Set S.Y., Yaguchi H., Tanaka Y., Jablonski M. (2004). Ultrafast fiber pulsed lasers incorporating carbon nanotubes. IEEE J. Sel. Top. Quantum Electron..

[34] Bao Q., Zhang H., Wang Y., Ni Z., Yan Y., Shen Z.X., Loh K.P., Tang D.Y. (2009). Atomic-layer graphene as a saturable absorber for ultrafast pulsed lasers. Adv. Funct. Mater..

[35] Zhang B., Liu J., Wang C., Yang K., Lee C., Zhang H., He J. (2020). Recent Progress in 2D Material-Based Saturable Absorbers for All Solid-State Pulsed Bulk Lasers. Laser Photonics Rev..

[36] Novoselov K.S., Geim A.K., Morozov S.V., Jiang D., Zhang Y., Dubonos S.V., Grigorieva I.V., Firsov A.A. (2004). Electric field effect in atomically thin carbon films. Science.

[37] Zhao C., Zhang H., Qi X., Chen Y., Wang Z., Wen S., Tang D. (2012). Ultra-short pulse generation by a topological insulator based saturable absorber. Appl. Phys. Lett..

[38] Salim M.A.M., Ab Razak M.Z., Azzuhri S.R., Ismail M.A. (2019). Generation of Microsecond Ytterbium-Doped Fibre Laser Pulses using Bismuth Telluride Thin Film as Saturable Absorber. Sains Malays..

[39] Zhang H., Lu S., Zheng J., Du J., Wen S., Tang D., Loh K. (2014). Molybdenum disulfide (MoS_2_) as a broadband saturable absorber for ultra-fast photonics. Opt. Express.

[40] Aizi Mat Salim M., Razalli Azzuhri S., Afiq Ismail M., Ab Razak M.Z. (2020). Few Layer Molybdenum Selenide Saturable Absorber using Optical Deposition Technique for Q-switched Ytterbium Pulses Laser Generation. J. Phys. Conf. Ser..

[41] Chen Y., Jiang G., Chen S., Guo Z., Yu X., Zhao C., Zhang H., Bao Q., Wen S., Tang D. (2015). Mechanically exfoliated black phosphorus as a new saturable absorber for both Q-switching and mode-locking laser operation. Opt. Express.

[42] Sotor J., Sobon G., Macherzynski W., Paletko P., Abramski K.M. (2015). Black phosphorus saturable absorber for ultrashort pulse generation. Appl. Phys. Lett..

[43] Jhon Y.I., Koo J., Anasori B., Seo M., Lee J.H., Gogotsi Y., Jhon Y.M. (2017). Metallic MXene saturable absorber for femtosecond mode-locked lasers. Adv. Mater..

[44] Song Y., Liang Z., Jiang X., Chen Y., Li Z., Lu L., Ge Y., Wang K., Zheng J., Lu S. (2017). Few-layer antimonene decorated microfiber: Ultra-short pulse generation and all-optical thresholding with enhanced long term stability. 2D Mater..

[45] Hu P., Liu Y., Guo L., Ge X., Liu X., Yu L., Liu Q. (2019). Passively Q-switched erbium-doped fiber laser based on antimonene as saturable absorber. Appl. Opt..

[46] Lu L., Liang Z., Wu L., Chen Y., Song Y., Dhanabalan S.C., Ponraj J.S., Dong B., Xiang Y., Xing F. (2018). Few-layer Bismuthene: Sonochemical Exfoliation, Nonlinear Optics and Applications for Ultrafast Photonics with Enhanced Stability. Laser Photonics Rev..

[47] Chai T., Li X., Feng T., Guo P., Song Y., Chen Y., Zhang H. (2018). Few-layer bismuthene for ultrashort pulse generation in a dissipative system based on an evanescent field. Nanoscale.

[48] Guo B., Wang S.-H., Wu Z.-X., Wang Z.-X., Wang D.-H., Huang H., Zhang F., Ge Y.-Q., Zhang H. (2018). Sub-200 fs soliton mode-locked fiber laser based on bismuthene saturable absorber. Opt. Express.

[49] Ma X., Zhang Z., Jiang W., Tong L., Liu S., Dai W., Chen W., Zhou Y., Zhang W., Qiu J. (2021). Passively mode-locked thulium doped fiber laser based on SnSe nanoparticles as a saturable absorber. Opt. Laser Technol..

[50] Chhowalla M., Shin H.S., Eda G., Li L.-J., Loh K.P., Zhang H. (2013). The chemistry of two-dimensional layered transition metal dichalcogenide nanosheets. Nat. Chem..

[51] Wilson J.A., Yoffe A. (1969). The transition metal dichalcogenides discussion and interpretation of the observed optical, electrical and structural properties. Adv. Phys..

[52] Li X., Chen C., Yang Y., Lei Z., Xu H. (2020). 2D Re-Based Transition Metal Chalcogenides: Progress, Challenges, and Opportunities. Adv. Sci..

[53] Rahman M., Davey K., Qiao S.Z. (2017). Advent of 2D rhenium disulfide (ReS_2_): Fundamentals to applications. Adv. Funct. Mater..

[54] Tongay S., Sahin H., Ko C., Luce A., Fan W., Liu K., Zhou J., Huang Y.-S., Ho C.-H., Yan J. (2014). Monolayer behaviour in bulk ReS_2_ due to electronic and vibrational decoupling. Nat. Commun..

[55] Zhang Q., Fu L. (2019). Novel Insights and Perspectives into Weakly Coupled ReS_2_ toward Emerging Applications. Chem.

[56] Zhang E., Jin Y., Yuan X., Wang W., Zhang C., Tang L., Liu S., Zhou P., Hu W., Xiu F. (2015). ReS_2_-Based Field-Effect Transistors and Photodetectors. Adv. Funct. Mater..

[57] Zulkefli A., Mukherjee B., Iwasaki T., Hayakawa R., Nakaharai S., Wakayama Y. (2020). Gate-bias tunable humidity sensors based on rhenium disulfide field-effect transistors. Jpn. J. Appl. Phys..

[58] Hao Q., Wang C., Liu W., Liu X., Liu J., Zhang H. (2020). Low-dimensional saturable absorbers for ultrafast photonics in solid-state bulk lasers: Status and prospects. Nanophotonics.

[59] Liu X., Gao Q., Zheng Y., Mao D., Zhao J. (2020). Recent progress of pulsed fiber lasers based on transition-metal dichalcogenides and black phosphorus saturable absorbers. Nanophotonics.

[60] Cui Y., Lu F., Liu X. (2017). Nonlinear Saturable and Polarization-induced Absorption of Rhenium Disulfide. Sci. Rep..

[61] Xu X., Jiang M., Li D., Wang R., Ren Z., Bai J. (2018). Passive Q-switching based on ReS_2_ saturable absorber in Er-doped fiber laser at 1532 nm. Opt Quantum Electron.

[62] Su X., Nie H., Wang Y., Li G., Yan B., Zhang B., Yang K., He J. (2017). Few-layered ReS_2_ as saturable absorber for 2.8 μm solid state laser. Opt. Lett..

[63] Mao D., Cui X., Gan X., Li M., Zhang W., Lu H., Zhao J. (2018). Passively Q-Switched and Mode-Locked Fiber Laser Based on an ReS_2_ Saturable Absorber. IEEE J. Sel. Top. Quantum Electron..

[64] He J., Zeng G., Liu S., Lu H., Xie R., Qi J., Tao L., Zhou B. (2021). Preparation of ultrathin ReS_2_ nanosheets and their application to Q-switched Er-doped fiber lasers. Front. Inf. Technol. Electron. Eng..

[65] Zhao R., Li G., Zhang B., He J. (2018). Multi-wavelength bright-dark pulse pair fiber laser based on rhenium disulfide. Opt. Express.

[66] Zhang M., Yin J., Yan P. Two-dimensional ReS_2_ nanosheets based saturable absorbers for passively mode-locked fiber lasers. Proceedings of the CLEO Pacific Rim Conference 2018.

[67] Xu X., He M., Quan C., Wang R., Liu C., Zhao Q., Zhou Y., Bai J., Xu X. (2018). Saturable Absorption Properties of ReS_2_ Films and Mode-Locking Application Based on Double-Covered ReS_2_ Micro Fiber. J. Light. Technol..

[68] Zuo C., Cao Y., Yang Q., He J., Zhang B. (2019). Passively Q-switched 2.95-μm bulk laser based on rhenium disulfide as saturable absorber. IEEE Photon. Technol. Lett..

[69] Liu S., Wang M., Yin S., Xie Z., Wang Z., Zhou S., Chen P. (2019). Nonlinear Optical Properties of Few-Layer Rhenium Disulfide Nanosheets and Their Passively Q-switched Laser Application. Phys. Status Solidi A.

[70] Lin M., Peng Q., Hou W., Fan X., Liu J. (2019). 1.3 μm Q-switched solid-state laser based on few-layer ReS_2_ saturable absorber. Opt. Laser Technol..

[71] Han S., Zhou S., Liu X., Liu Y., Zhang S., Yang X. (2018). Rhenium disulfide-based passively Q-switched dual-wavelength laser at 0.95 μm and 1.06 μm in Nd:YAG. Laser Phys. Lett..

[72] Noddack W. (1925). Die ekamangane. Naturwissenschaften.

[73] Lin Y.-C., Komsa H.-P., Yeh C.-H., Bjorkman T., Liang Z.-Y., Ho C.-H., Huang Y.-S., Chiu P.-W., Krasheninnikov A.V., Suenaga K. (2015). Single-layer ReS_2_: Two-dimensional semiconductor with tunable in-plane anisotropy. ACS Nano.

[74] Liu E., Fu Y., Wang Y., Feng Y., Liu H., Wan X., Zhou W., Wang B., Shao L., Ho C.-H. (2015). Integrated digital inverters based on two-dimensional anisotropic ReS_2_ field-effect transistors. Nat. Commun..

[75] Yun W.S., Han S., Hong S.C., Kim I.G., Lee J. (2012). Thickness and strain effects on electronic structures of transition metal dichalcogenides: 2H-M X 2 semiconductors (M = Mo, W; X = S, Se, Te). Phys. Rev. B.

[76] Zhang H., Li X.-B., Liu L.-M. (2013). Tunable electronic and magnetic properties of WS2 nanoribbons. J. Appl. Phys..

[77] Ho C., Huang Y., Tiong K., Liao P. (1999). In-plane anisotropy of the optical and electrical properties of layered ReS_2_ crystals. J. Phys. Condens. Matter.

[78] Mohamed N.B., Shinokita K., Wang X., Lim H.E., Tan D., Miyauchi Y., Matsuda K. (2018). Photoluminescence quantum yields for atomically thin-layered ReS2: Identification of indirect-bandgap semiconductors. Appl. Phys. Lett..

[79] Chenet D.A., Aslan O.B., Huang P.Y., Fan C., van der Zande A.M., Heinz T.F., Hone J.C. (2015). In-Plane Anisotropy in Mono- and Few-Layer ReS2 Probed by Raman Spectroscopy and Scanning Transmission Electron Microscopy. Nano Lett..

[80] Feng Y., Zhou W., Wang Y., Zhou J., Liu E., Fu Y., Ni Z., Wu X., Yuan H., Miao F. (2015). Raman vibrational spectra of bulk to monolayer ReS2 with lower symmetry. Phys. Rev. B.

[81] Nagler P., Plechinger G., Schüller C., Korn T. (2016). Observation of anisotropic interlayer Raman modes in few-layer ReS_2_. Phys. Status Solidi RRL.

[82] Lee C., Yan H., Brus L.E., Heinz T.F., Hone J., Ryu S. (2010). Anomalous Lattice Vibrations of Single- and Few-Layer MoS_2_. ACS Nano.

[83] Lee C., Wei X., Kysar J.W., Hone J. (2008). Measurement of the elastic properties and intrinsic strength of monolayer graphene. Science.

[84] Bertolazzi S., Brivio J., Kis A. (2011). Stretching and Breaking of Ultrathin MoS_2_. ACS Nano.

[85] Novoselov K.S., Geim A.K., Morozov S.V., Jiang D., Katsnelson M.I., Grigorieva I.V., Dubonos S.V., Firsov A.A. (2005). Two-dimensional gas of massless Dirac fermions in graphene. Nature.

[86] Zhang Y., Tan Y.-W., Stormer H.L., Kim P. (2005). Experimental observation of the quantum Hall effect and Berry’s phase in graphene. Nature.

[87] Liu E., Long M., Zeng J., Luo W., Wang Y., Pan Y., Zhou W., Wang B., Hu W., Ni Z. (2016). High responsivity phototransistors based on few-layer ReS_2_ for weak signal detection. Adv. Funct. Mater..

[88] Lorchat E., Froehlicher G., Berciaud S. (2016). Splitting of Interlayer Shear Modes and Photon Energy Dependent Anisotropic Raman Response in N-Layer ReSe2 and ReS_2_. ACS Nano.

[89] Xiong Y., Chen H., Zhang D.W., Zhou P. (2019). Electronic and optoelectronic applications based on ReS_2_. Phys. Status Solidi—Rapid Res. Lett..

[90] Kang J., Sangwan V.K., Wood J.D., Liu X., Balla I., Lam D., Hersam M.C. (2016). Layer-by-Layer Sorting of Rhenium Disulfide via High-Density Isopycnic Density Gradient Ultracentrifugation. Nano Lett..

[91] Xu X., Guo Y., Zhao Q., Si K., Zhou Y., Ma J., Bai J., Xu X. (2018). Green and efficient exfoliation of ReS_2_ and its photoelectric response based on electrophoretic deposited photoelectrodes. Mater. Des..

[92] Miao Z.H., Lv L.X., Li K., Liu P.Y., Li Z., Yang H., Zhao Q., Chang M., Zhen L., Xu C.Y. (2018). Liquid exfoliation of colloidal rhenium disulfide nanosheets as a multifunctional theranostic agent for in vivo photoacoustic/ct imaging and photothermal therapy. Small.

[93] Fujita T., Ito Y., Tan Y., Yamaguchi H., Hojo D., Hirata A., Voiry D., Chhowalla M., Chen M. (2014). Chemically exfoliated ReS_2_ nanosheets. Nanoscale.

[94] Wu S., Huang C., Aivazian G., Ross J.S., Cobden D.H., Xu X. (2013). Vapor–solid growth of high optical quality MoS2 monolayers with near-unity valley polarization. ACS Nano.

[95] Feng Q., Zhu Y., Hong J., Zhang M., Duan W., Mao N., Wu J., Xu H., Dong F., Lin F. (2014). Growth of Large-Area 2D MoS2(1-x)Se2x Semiconductor Alloys. Adv. Mater..

[96] Qi F., Chen Y., Zheng B., Zhou J., Wang X., Li P., Zhang W. (2016). Facile growth of large-area and high-quality few-layer ReS_2_ by physical vapour deposition. Mater. Lett..

[97] Wang D., Luo F., Lu M., Xie X., Huang L., Huang W. (2019). Chemical Vapor Transport Reactions for Synthesizing Layered Materials and Their 2D Counterparts. Small.

[98] Jariwala B., Voiry D., Jindal A., Chalke B.A., Bapat R., Thamizhavel A., Chhowalla M., Deshmukh M., Bhattacharya A. (2016). Synthesis and Characterization of ReS_2_ and ReSe_2_ Layered Chalcogenide Single Crystals. Chem. Mater..

[99] Xing L., Yan X., Zheng J., Xu G., Lu Z., Liu L., Wang J., Wang P., Pan X., Jiao L. (2019). Highly crystalline ReSe_2_ atomic layers synthesized by chemical vapor transport. InfoMat.

[100] Zhou X., Gan L., Tian W., Zhang Q., Jin S., Li H., Bando Y., Golberg D., Zhai T. (2015). Ultrathin SnSe_2_ Flakes Grown by Chemical Vapor Deposition for High-Performance Photodetectors. Adv. Mater..

[101] Lee Y.-H., Zhang X.-Q., Zhang W., Chang M.-T., Lin C.-T., Chang K.-D., Yu Y.-C., Wang J.T.-W., Chang C.-S., Li L.-J. (2012). Synthesis of Large-Area MoS_2_ Atomic Layers with Chemical Vapor Deposition. Adv. Mater..

[102] Keyshar K., Gong Y., Ye G., Brunetto G., Zhou W., Cole D.P., Hackenberg K., He Y., Machado L., Kabbani M. (2015). Chemical vapor deposition of monolayer rhenium disulfide (ReS_2_). Adv. Mater..

[103] Hafeez M., Gan L., Li H., Ma Y., Zhai T. (2016). Large-area bilayer ReS_2_ film/multilayer ReS_2_ flakes synthesized by chemical vapor deposition for high performance photodetectors. Adv. Funct. Mater..

[104] Dathbun A., Kim Y., Kim S., Yoo Y., Kang M.S., Lee C., Cho J.H. (2017). Large-Area CVD-Grown Sub-2 V ReS_2_ Transistors and Logic Gates. Nano Lett..

[105] Yin J., Li J., Chen H., Wang J., Yan P., Liu M., Liu W., Lu W., Xu Z., Zhang W. (2017). Large-area highly crystalline WSe_2_ atomic layers for ultrafast pulsed lasers. Opt. Express.

[106] Wang G., Baker-Murray A.A., Blau W.J. (2019). Saturable Absorption in 2D Nanomaterials and Related Photonic Devices. Laser Photonics Rev..

[107] Liu W., Liu M., Liu X., Wang X., Deng H.X., Lei M., Wei Z., Wei Z. (2020). Recent advances of 2D materials in nonlinear photonics and fiber lasers. Adv. Opt. Mater..

[108] Du J., Wang Q., Jiang G., Xu C., Zhao C., Xiang Y., Chen Y., Wen S., Zhang H. (2014). Ytterbium-doped fiber laser passively mode locked by few-layer Molybdenum Disulfide (MoS_2_) saturable absorber functioned with evanescent field interaction. Sci. Rep..

[109] Song Y.-W., Yamashita S., Maruyama S. (2008). Single-walled carbon nanotubes for high-energy optical pulse formation. Appl. Phys. Lett..

[110] Ge Y., Zhu Z., Xu Y., Chen Y., Chen S., Liang Z., Song Y., Zou Y., Zeng H., Xu S. (2018). Broadband Nonlinear Photoresponse of 2D TiS_2_ for Ultrashort Pulse Generation and All-Optical Thresholding Devices. Adv. Opt. Mater..

[111] Khazaeinezhad R., Hosseinzadeh Kassani S., Nazari T., Jeong H., Kim J., Choi K., Lee J.-U., Kim J.H., Cheong H., Yeom D.-I. (2015). Saturable optical absorption in MoS_2_ nano-sheet optically deposited on the optical fiber facet. Opt. Commun..

[112] Ahmad H., Muhammad F.D., Zulkifli M.Z., Harun S.W. (2012). Graphene-Oxide-Based Saturable Absorber for All-Fiber Q-Switching with a Simple Optical Deposition Technique. IEEE Photonics J..

[113] Jin X., Hu G., Zhang M., Hu Y., Albrow-Owen T., Howe R.C., Wu T.-C., Wu Q., Zheng Z., Hasan T. (2018). 102 fs pulse generation from a long-term stable, inkjet-printed black phosphorus-mode-locked fiber laser. Opt. Express.

[114] Zhang M., Wu Q., Zhang F., Chen L., Jin X., Hu Y., Zheng Z., Zhang H. (2019). 2D Black Phosphorus Saturable Absorbers for Ultrafast Photonics. Adv. Opt. Mater..

[115] Mao D., Li M., Cui X., Zhang W., Lu H., Song K., Zhao J. (2018). Stable high-power saturable absorber based on polymer-black-phosphorus films. Opt. Commun..

[116] Sheik-Bahae M., Said A.A., Wei T., Hagan D.J., Stryland E.W.V. (1990). Sensitive measurement of optical nonlinearities using a single beam. IEEE J. Quantum Electron..

[117] Woodward R., Howe R., Hu G., Torrisi F., Zhang M., Hasan T., Kelleher E. (2015). Few-layer MoS_2_ saturable absorbers for short-pulse laser technology: Current status and future perspectives. Photonics Res..

[118] Jeon J., Lee J., Lee J.H. (2015). Numerical study on the minimum modulation depth of a saturable absorber for stable fiber laser mode locking. JOSA B.

[119] Haiml M., Grange R., Keller U. (2004). Optical characterization of semiconductor saturable absorbers. Appl. Phys. B.

[120] Zhang Y., Lu D., Yu H., Zhang H. (2019). Low-Dimensional Saturable Absorbers in the Visible Spectral Region. Adv. Opt. Mater..

[121] Du J., Zhang M., Guo Z., Chen J., Zhu X., Hu G., Peng P., Zheng Z., Zhang H. (2017). Phosphorene quantum dot saturable absorbers for ultrafast fiber lasers. Sci. Rep..

[122] Guo B. (2018). 2D noncarbon materials-based nonlinear optical devices for ultrafast photonics. Chin. Opt. Lett..

[123] Su X., Zhang B., Wang Y., He G., Li G., Lin N., Yang K., He J., Liu S. (2018). Broadband rhenium disulfide optical modulator for solid-state lasers. Photonics Res..

[124] Zhang N., Zeng Z., Wang Z., Li B., Pan Y. (2020). Nd:YSAG Q-switched laser with anisotropic ReS_2_ nanosheets. Optik.

[125] Fan M., Li T., Zhao J., Zhao S., Li G., Yang K., Su L., Ma H., Kränkel C. (2018). Continuous wave and ReS_2_ passively Q-switched Er : SrF_2_ laser at ∼3  μm. Opt. Lett..

[126] Lu B., Wen Z., Huang K., Qi X., Wang N., Chen H., Bai J. (2019). Passively Q-Switched Yb^3+^-Doped Fiber Laser With ReS_2_ Saturable Absorber. IEEE J. Sel. Top. Quantum Electron..

[127] Zhang S., Ma Y., Liu X., Ding S., Yu X., Zhang Q. (2020). Continuous wave and rhenium disulfide passively Q-switched Nd:GdLaNbO_4_ laser under direct pumping. Opt. Commun..

[128] Zhou Y., Fang C., Zhang Z., Tong L., Ma X., Zhang W., Yu R., Gao W., Xu J., Liao M. (2020). Sub-picosecond passively mode-locked thulium-doped fiber laser by ReS_2_ nanoparticles. Jpn. J. Appl. Phys..

[129] Steinberg D., Zapata J.D., Souza E.A.T.d., Saito L.A.M. Mechanically exfoliated Rhenium disulfide onto D-shaped optical fiber for sub-300 fs EDFL mode-locking. Proceedings of the 2018 Conference on Lasers and Electro-Optics (CLEO).

[130] Cui Q., He J., Bellus M.Z., Mirzokarimov M., Hofmann T., Chiu H.Y., Antonik M., He D., Wang Y., Zhao H. (2015). Transient absorption measurements on anisotropic monolayer ReS_2_. Small.

[131] Trushin M., Kelleher E.J.R., Hasan T. (2016). Theory of edge-state optical absorption in two-dimensional transition metal dichalcogenide flakes. Phys. Rev. B.

[132] Mao D., Zhang S., Wang Y., Gan X., Zhang W., Mei T., Wang Y., Wang Y., Zeng H., Zhao J. (2015). WS_2_ saturable absorber for dissipative soliton mode locking at 1.06 and 1.55 µm. Opt. Express.

[133] Wang S., Yu H., Zhang H., Wang A., Zhao M., Chen Y., Mei L., Wang J. (2014). Broadband few-layer MoS_2_ saturable absorbers. Adv. Mater..

[134] Woodward R.I., Kelleher E.J.R., Howe R.C.T., Hu G., Torrisi F., Hasan T., Popov S.V., Taylor J.R. (2014). Tunable Q-switched fiber laser based on saturable edge-state absorption in few-layer molybdenum disulfide (MoS2). Opt. Express.

[135] Wang K., Feng Y., Chang C., Zhan J., Wang C., Zhao Q., Coleman J.N., Zhang L., Blau W.J., Wang J. (2014). Broadband ultrafast nonlinear absorption and nonlinear refraction of layered molybdenum dichalcogenide semiconductors. Nanoscale.

[136] Horzum S., Çakır D., Suh J., Tongay S., Huang Y.-S., Ho C.-H., Wu J., Sahin H., Peeters F. (2014). Formation and stability of point defects in monolayer rhenium disulfide. Phys. Rev. B.

[137] Karatay A., Yaglioglu H.G., Elmali A., Parlak M., Karaagac H. (2012). Thickness-dependent nonlinear absorption behaviors in polycrystalline ZnSe thin films. Opt. Commun..

[138] Yu Z., Song Y., Tian J., Dou Z., Guoyu H., Li K., Li H., Zhang X. (2014). High-repetition-rate Q-switched fiber laser with high quality topological insulator Bi_2_Se_3_ film. Opt. Express.

[139] Li H., Xia H., Lan C., Li C., Zhang X., Li J., Liu Y. (2014). Passively Q-switched erbium-doped fiber laser based on few-layer MoS_2_ saturable absorber. IEEE Photonics Technol. Lett..

[140] Cao Y.-D., Sun Y.-H., Shi S.-F., Wang R.-M. (2021). Anisotropy of two-dimensional ReS_2_ and advances in its device application. Rare Met..

[141] Wang Z., Zhang B., Liu J., Song Y., Zhang H. (2020). Recent developments in mid-infrared fiber lasers: Status and challenges. Opt. Laser Technol..

[142] Liu L., Chu H., Zhang X., Pan H., Zhao S., Li D. (2019). Heterostructure ReS_2_/GaAs Saturable Absorber Passively Q-Switched Nd:YVO4 Laser. Nanoscale Res. Lett..

[143] Ahn J., Kyhm J.-H., Kang H.K., Kwon N., Kim H.-K., Park S., Hwang D.K. (2021). 2D MoTe_2_/ReS_2_ van der Waals Heterostructure for High-Performance and Linear Polarization-Sensitive Photodetector. ACS Photonics.

[144] Wadhwa R., Agrawal A.V., Kushavah D., Mushtaq A., Pal S.K., Kumar M. (2021). Investigation of charge transport and band alignment of MoS_2_-ReS_2_ heterointerface for high performance and self-driven broadband photodetection. Appl. Surf. Sci..

[145] Wang Z., Zeng P., Hu S., Wu X., He J., Wu Z., Wang W., Zheng P., Zheng H., Zheng L. (2021). Broadband photodetector based on ReS_2_/graphene/WSe_2_ heterostructure. Nanotechnology.

